# Direct Integration of Spent LiMn_2_O_4_ with High Voltage Aqueous Zinc‐Manganese Redox Flow Batteries as a Practical Upcycling Process

**DOI:** 10.1002/smll.202500787

**Published:** 2025-03-11

**Authors:** Duho Han, Hyeokjun Jang, Jinyeong Choi, Jihan Park, Seongmin Kim, Seongsoo Han, Wonjae Lee, Jin Hong Lee, Pilgun Oh, Yosep Han, Minjoon Park

**Affiliations:** ^1^ Department of Nanoenergy Engineering Pusan National University 50, Busan daehak‐ro 63 beon‐gil 2 Busan Geumjeong‐gu 46241 Republic of Korea; ^2^ Department of Nano Fusion Technology Pusan National University Busandaehak‐ro 63beon‐gil 2 Busan Geumjeong‐gu 46241 Republic of Korea; ^3^ Research Center of Energy Convergence Technology Pusan National University Busandaehak‐ro 63beon‐gil 2 Busan Geumjeong‐gu 46241 Republic of Korea; ^4^ School of Chemical Engineering Pusan National University Busan Geumjeong‐gu 46241 Republic of Korea; ^5^ Department of Smart Green Technology Engineering Department of Nanotechnology Engineering Pukyong National University 45, Yongso‐ro, Nam‐gu Busan 48547 Republic of Korea; ^6^ Resources Utilization Division Korea Institute of Geoscience and Mineral Resources (KIGAM) Daejeon 34132 Republic of Korea

**Keywords:** battery recycling, battery upcycling, electrochemical reactions, energy storage systems, lithium manganese oxide, redox flow batteries (RFBs)

## Abstract

With the explosive growth of lithium‐ion batteries (LIBs), research on the recycling of spent batteries is widely conducted. However, conventional processes involve complex procedures, high costs, and environmental issues. This study introduces the electrochemical upcycling of spent LiMn_2_O_4_ (LMO) cathode material, incorporating pre‐filtration (PF) and pre‐reduction (PR) processes to enable its direct application in redox flow batteries (RFBs). Moreover, a double membrane system is applied to address the low operating voltage and energy density by balancing the different pH levels of the zinc anode and manganese cathode. The aqueous zinc‐manganese RFB, derived from spent LMO pouch full cells, achieves coulombic efficiency of 90% and energy efficiency (EE) exceeding 70% over 250 cycles. The LMO‐containing electrolyte is further treated to precipitate the manganese ions by a simple pH adjustment, enabling 100% separation of lithium, thereby enhancing the sustainability of rare metal resources. This work presents a remarkable advancement in the upcycling method of LIB cathode materials and contributes to establishing a circular system for battery materials.

## Graphical abstract

To address the high energy consumption and complex processes of conventional battery recycling, a novel upcycling strategy is proposed, where spent cathode materials undergo filtration and electrochemical reduction reactions using carbon felt (CF) electrodes in redox flow batteries (RFBs). After use, the catholyte is processed to separate its constituent metals, achieving closed‐loop recycling.

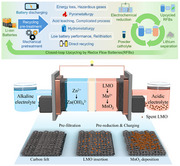

## 1 Introduction

Lithium‐ion batteries (LIBs) have become the primary power source for portable electronic devices, electric vehicles, and large‐scale energy storage systems due to their high energy density, long cycle life, and lightweight characteristics.^[^ ^1^, ^2^ ^]^ However, the rapid proliferation of LIBs poses significant environmental and resource challenges.^[^ ^3^ ^]^ With the increasing adoption of electric vehicles and energy storage systems, the usage of LIBs is expected to continue growing.^[^ ^4^, ^5^, ^6^ ^]^ These spent cathode materials contain valuable metals such as cobalt, nickel, manganese, and lithium.^[^ ^7^ ^]^ Therefore, the proper treatment and recycling of spent batteries are crucial not only to mitigate environmental pollution and health risks, but also to ensure a sustainable supply of strategic resources.

Conventional recycling methods for LIBs, such as pyrometallurgical and hydrometallurgical processes,^[^ ^8^ ^]^ are energy‐intensive, environmentally problematic, and involve complex procedures. Another major issue is that these methods primarily target the high‐value cathodes like LiCoO_2_ (LCO) and LiNi_x_Co_y_Mn_z_O_2_ (NCM),^[^ ^9^, ^10^ ^]^ while lower‐cost cathodes such as LiMn_2_O_4_ (LMO) are often neglected owing to their lower economic value. Existing studies on LMO recycling still rely on high‐temperature processes or involve complex procedures. The LMO is widely used in large‐scale energy storage applications because of its relatively high operating voltage, low cost, high safety, and environmental friendliness.^[^ ^11^ ^]^ Over time, the amount of LMO waste will continue to increase. Therefore, there is an urgent necessity to develop efficient, economical strategies for recycling the LMO.^[^ ^12^, ^13^ ^]^ Moreover, the direct recycling has recently gained attention as it involves the direct reuse of active materials in batteries.^[^ ^14^, ^15^, ^16^ ^]^ By avoiding high‐temperature treatment and complex acid‐leaching processes, the direct recycling is considered a more efficient approach, maximizing the recovery value of cathode materials. Consequently, various regeneration methods, such as hydrothermal treatment,^[^ ^17^ ^]^ electrochemical relithiation, and chemical relithiation,^[^ ^18^ ^]^ are being investigated,^[^ ^19^ ^]^ though they are mainly focused on expensive metals like cobalt and nickel. In contrast, there is still a lack of research on recycling promising materials such as NCM613 and LMO, which exhibit high‐power characteristics.

Furthermore, several efforts have been made to convert spent electrodes into high‐value products, also known as upcycling. The metals from the cathode materials are upcycled based on the aforementioned recycling methods, contributing to the sustainable development of the spent LIB recycling industry. E.g., Miaomiao et al. reported an upcycled cathode material by doping Mn at the Co site and N and S between the Li‐O sites of degraded LiCoO_2_.^[^ ^20^ ^]^ Although great progress has been achieved, the upcycling process involves complex procedures and is still largely limited to doping from a materials perspective. In addition, as global reliance on renewable energy sources such as solar and wind continues to rise, the need for efficient and scalable energy storage systems has never been greater.^[^ ^21^ ^]^ Aqueous redox flow batteries (RFBs) are promising technologies for integrating these intermittent energy sources into the power grid. They are inherently safe, employing non‐flammable electrolytes, and their power output and capacity can be independently scaled, making them suitable for large‐scale energy storage.^[^ ^22^ ^]^ In the context of aqueous RFBs, integrating cathode materials such as manganese recycled from the LMO represents a novel approach to improve the system sustainability and cost‐effectiveness.

A primary drawback of conventional RFBs is their low operating voltage and energy density. Therefore, it is essential to select an appropriate redox couple for the anode and cathode and optimize the system configuration.^[^ ^23^, ^24^, ^25^ ^]^ For instance, Walid A. Daoud et al. introduced a zinc anode and cerium cathode with a dual membrane system, achieving a high discharge voltage of 2.3 V and an energy efficiency (EE) of 71.3%.^[^ ^26^ ^]^ Specifically, RFBs based on a zinc anode and manganese cathode combine the low cost of manganese with the excellent electrochemical properties of zinc,^[^ ^27^, ^28^ ^]^ enhancing the operating voltage of the system,^[^ ^29^ ^]^ reducing dependence on expensive metals, and minimizing environmental impact.^[^ ^30^, ^31^, ^32^ ^]^

In this study, we introduced a hybrid electrochemical upcycling approach that applies recycled LMO cathode materials in a Zinc‐LMO hybrid RFB system. Unlike pyrometallurgical methods that require high temperatures and produce high CO_2_ emissions, or hydrometallurgical processes that generate hazardous chemical waste, our method operates as electrolytes, eliminating the need for extreme heat or energy.^[^ ^33^ ^]^ This hybrid process combines the advantages of hydrometallurgical treatment and direct recycling, simplifying conventional recycling procedures. Additionally, the manganese extracted from LMO enables high‐voltage operation in acidic electrolytes, offering an alternative to conventional MnSO_4_ catholyte. Instead of relying on complex separation and refining steps, our system directly utilizes Mn ions recovered from spent LMO through a double‐membrane configuration. An anion exchange membrane at the cathode and a cation exchange membrane at the anode effectively balance pH differences between the acidic manganese electrolyte and the alkaline zinc electrolyte, improving system stability.

The spent LMO undergoes electrochemical discharge in sulfuric acid, allowing for the controlled release of Mn and Li ions. The recovered manganese ions serve as active materials in the RFB, maintaining strong electrochemical performance while reducing environmental impact and process costs. This upcycling approach minimizes waste, lowers reagent consumption, and eliminates unnecessary refining, making it a sustainable and cost‐effective alternative to conventional LIB recycling. By directly integrating recycled LMO into the RFB system, this study offers an innovative, scalable solution for LIB recycling. Through this strategy, we contribute to the development of high‐energy‐density, low‐cost aqueous RFBs, supporting a circular economy and ensuring sustainable resource utilization.

## 2 Results and Discussion

### 2.1 Challenges of Conventional Recycling Methods and Novel Preparation Process for Upcycling Spent LMO Cathode Material

**Figure 1:**
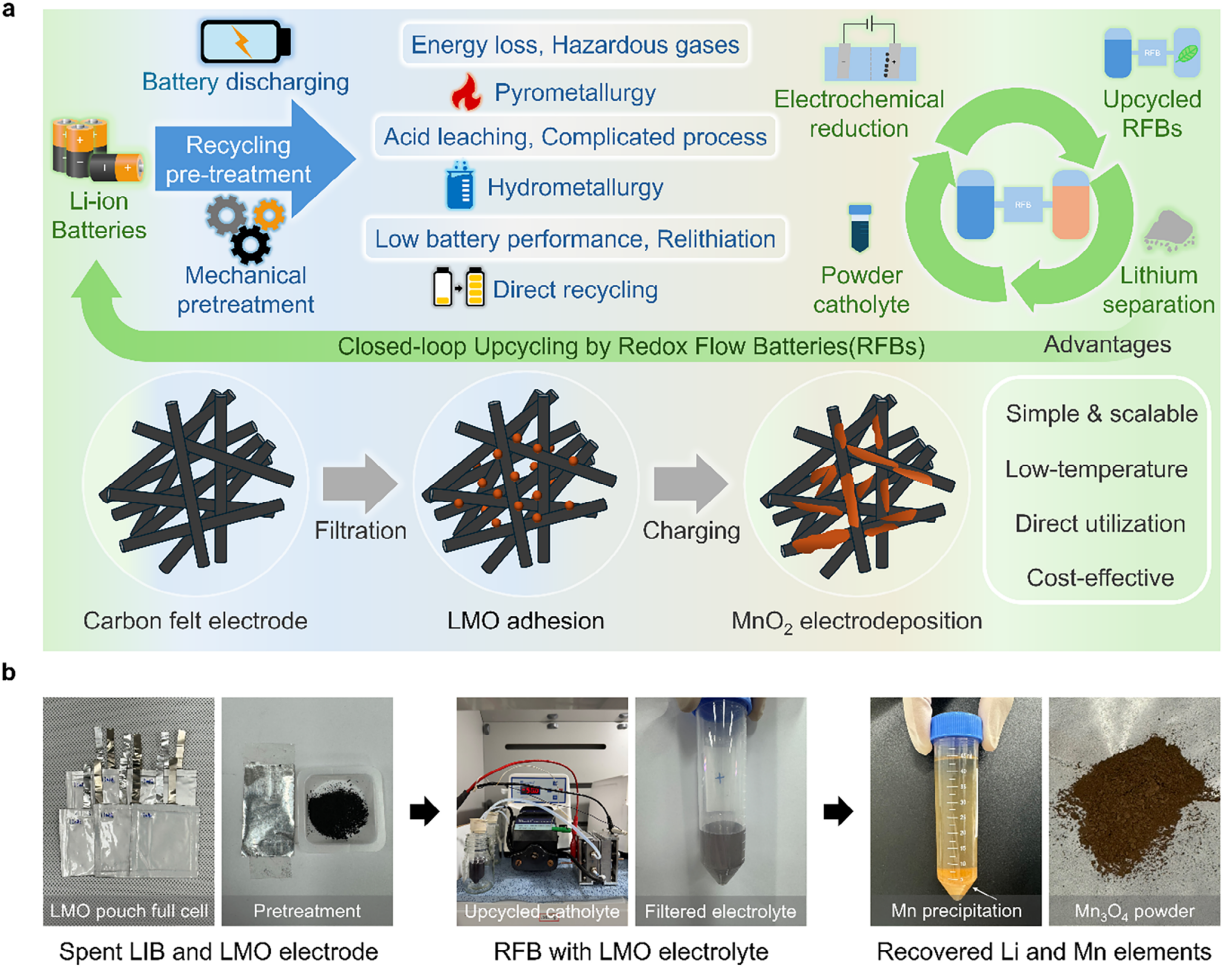
Comparison of conventional recycling methods and upcycling strategy. Schematic of the a) closed‐loop upcycling by RFBs and b) digital images of closed‐loop RFB upcycling process.

Conventional recycling methods face several challenges (**Figure** **1**a). LIBs typically undergo pre‐treatment processes such as battery discharging and mechanical pretreatment. Following this, they are subjected to one of three primary recycling approaches: pyrometallurgy, hydrometallurgy, or direct recycling. The pyrometallurgy recycling involves melting materials at temperatures exceeding 1000 °C, consuming excessive thermal energy and releasing toxic gases during combustion. The hydrometallurgy process dissolves materials in acidic or alkaline solutions, recovering metals through leaching and precipitation processes. However, this method generates large quantities of wastewater, raising environmental concerns, and involves complex procedures. Direct recycling focuses on reactivating cathode materials through processes such as relithiation. Despite its potential, this approach suffers from limitations in performance and applicability. Thus, we introduced an upcycling strategy utilizing the electrochemical reactions of RFBs. The LMO was electrochemically reacted at the RFB electrode and utilized as a manganese‐based catholyte. Through upcycling via the RFB, we aim to propose a simple yet scalable, low‐temperature, and cost‐effective strategy. Figure 1b presents a digital illustration of our process designed to realize closed‐loop upcycling. Spent LMO pouch cells undergo battery discharging and scrapping pretreatment, followed by electrochemical reduction within the RFB electrodes. Afterward, the used electrolyte is subjected to a pH adjustment process to separate Li and Mn ions. The Li‐ion solution can then be further processed and utilized as precursors for LIBs.

### 2.2 Design and Mechanism of the Aqueous Zn‐LMO Hybrid RFB with Pre‐Reduction (PR) and Redox Processes

**Figure 2:**
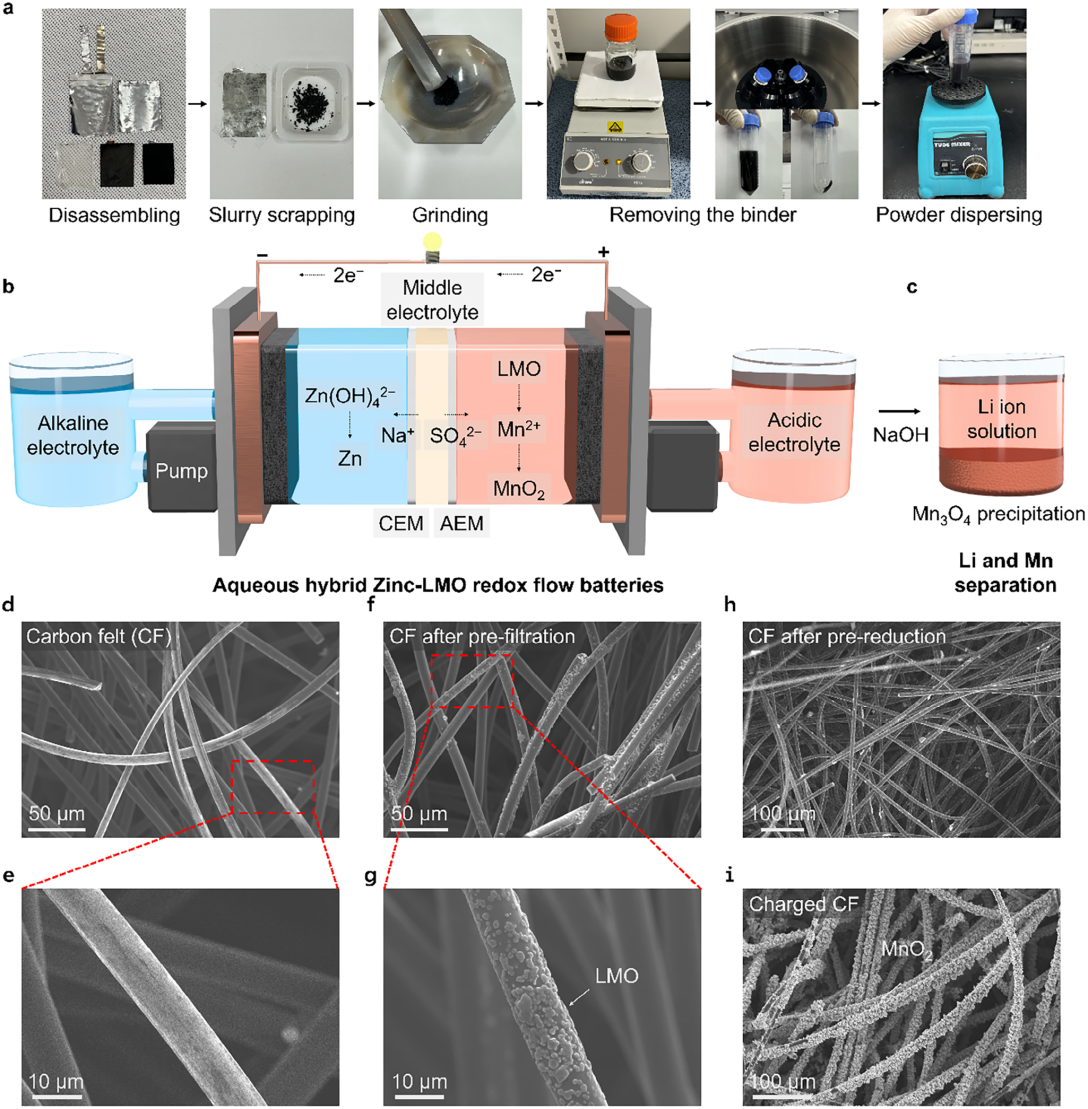
Aqueous hybrid Zinc‐LMO RFB schematic and surface morphology of CF electrode during the process. a) Digital images of fabricating LMO catholyte. b) A schematic of aqueous hybrid Zn‐LMO RFBs with an LMO catholyte, showing PR, charging, and c) Li separation process. The surface morphology characteristics of the d,e) pristine CF and f,g) after the PF. The SEM images of CF after the h) PF and i) charging process.

We introduced a novel preparation process for upcycling the spent LMO cathode material, which could be applied as the positive electrolyte in an RFB (**Figure** **2**a; Figure S1, Supporting Information). To ensure sufficient active material and practical application, the cathode material was recovered from the spent LMO pouch full cells. The spent LMO pouch cells, which had degraded to 80% of their initial capacity, were discharged and disassembled to eliminate the fire hazards. The discharged cells were carefully disassembled, and only the cathode slurry containing Li and Mn was scraped off and recovered. Organic electrolyte and Li salts in the scrapped slurry were considered contaminants in aqueous RFB systems, so they were washed away. The recovered cathode powder contained not only the active material, but also conductive agents and binders. The binders could obstruct the flow in aqueous systems, leading to blockages in pumps or channels. Therefore, after grinding, the powder was placed in an organic solvent (1‐Methyl‐2‐pyrrolidinone) to separate the binder, leaving only the active material and conductive agent in the powder. The powder was then dispersed in 2.5 m H_2_SO_4_ solution, which was used as the supporting electrolyte for the positive electrolyte, completing the preparation of the positive electrolyte. The dispersed powder flowed through the system, passing through the carbon felt (CF) electrode, which was filtered and remained within the felt. We termed this the process pre‐filtration (PF), and the spent LMO was separated from the LIB pouch cells for electrochemical reactions. Figure 2b shows a schematic of the aqueous Zn‐LMO hybrid RFB. To achieve a high‐voltage flow battery, an alkaline solution was applied to the anode and an acidic solution to the cathode.^[^ ^30^ ^]^ The pH difference between alkaline anolyte and acidic catholyte was managed by a double membrane system. A cation exchange membrane was used at the anode side, and an anion exchange membrane at the cathode side, with a neutral Na_2_SO_4_ solution as the middle electrolyte to prevent the crossover of hydrogen and hydroxide ions from the opposite sides. The cell voltage was compared with that of a conventional single membrane system (Figure S2, Supporting Information). While the neutral system using NaCl as the supporting electrolyte exhibited an average discharge voltage of ≈1.5 V, the hybrid system with 4.0 m NaOH and 2.5 m H_2_SO_4_ showed an average discharge voltage of ≈2.5 V. The filtered LMO powder underwent the PR process reactions, being reduced into Li and Mn^2+^ ions in the electrolyte (Equation 1). Subsequently, the reduced Mn ions were oxidized to MnO_2_ during charging (Equation 2). The mechanism of the PR process and redox couples in the aqueous Zn‐LMO hybrid RFB was illustrated in Figure S3 (Supporting Information).

Li ions, which did not participate in the redox reactions, remained in the electrolyte as ions after battery operation. To utilize the Li ions in the spent electrolyte, we introduced a simple process by adding NaOH solution to adjust the pH to alkaline (Figure 2c). Simply increasing the pH to alkaline conditions allowed the manganese oxides to precipitate as Mn_3_O_4_, resulting in a solution with only dissolved Li ions, which will be discussed later.

Scanning electron microscopy (SEM) analysis was conducted to observe differences at each stage of the prepared LMO dispersed electrolyte. The analysis showed the CF electrode before and after the PF and PR processes. Figure 2d,e shows the clean surface of the felt before the PF process. The electrolyte containing the dispersed LMO flowed without applying a current before starting the PR process (Figure S4, Supporting Information). Figure 2f shows the CF after the electrolyte had flowed through it. The result showed that the LMO powder adhered to the surface of the CF during the electrolyte flow. Also, the enlarged image (Figure 2g) confirmed that the LMO particles were evenly distributed on the CF surface. The powder was filtered, and no powder exited through the outlet, indicating that it adhered and remained within the CF. Observing the color of the electrolyte after the electrolyte flow, the solution appeared transparent (Figure S5, Supporting Information). This result suggested that the CF effectively captured the LMO particles and immobilized them on the CF electrode surface. After the electrolyte flow was completed, the PR process was performed at a low current density of 5 mA cm^−2^. The image taken after discharging to 1.0 V showed that all LMO particles on the CF surface had been electrochemically reduced, which returned to a surface similar to that before the electrolyte flow (Figure 2h; Figure S6, Supporting Information). Once the PR process was completed, Li and Mn elements were presented as metal ions in the sulfuric acid electrolyte, and during charging, Mn^2+^ was oxidized to solid MnO_2_, which was electrodeposited onto the CF (Figure 2i). When compared to the charging of the conventional MnSO_4_ electrolyte, similar MnO_2_ morphology was formed (Figure S7, Supporting Information). Energy dispersive X‐ray spectroscopy (EDS) mapping analysis also confirmed that the same charging reaction occurred by detecting the composition of the Mn and O species (Figures S8 and S9, Supporting Information).

### 2.3 Characterization of Charged Product Formation

**Figure 3:**
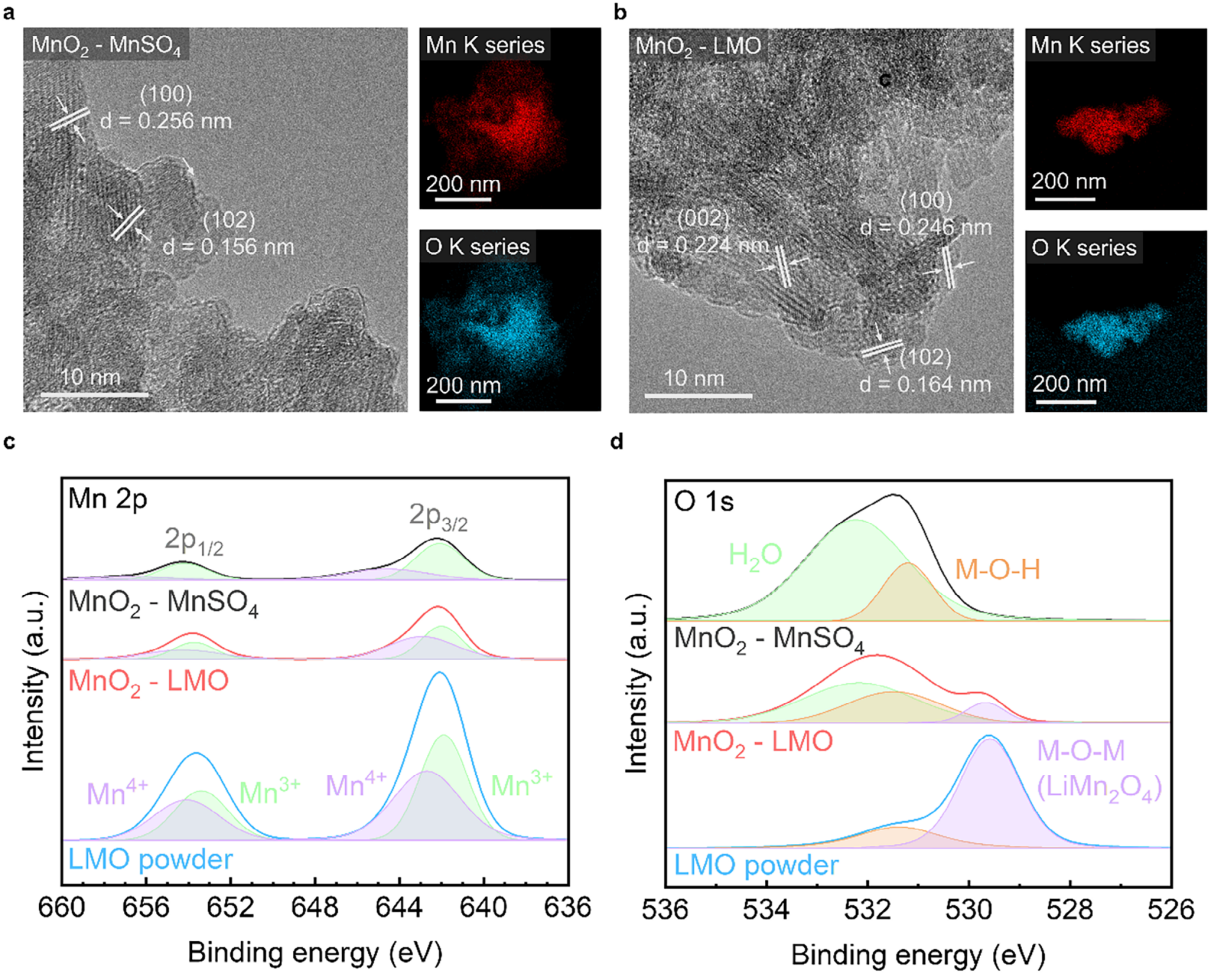
HR‐TEM image of a) the MnO_2_ charged from MnSO_4_ (P‐MnO_2_) and b) the MnO_2_ charged from the PR‐LMO (PR‐MnO_2_). The XPS spectra of the MnO_2_ from MnSO_4_ electrolyte (black line), from LMO electrolyte (red line), and LMO powder (blue line) for c) Mn 2p and d) O 1s.

High‐resolution transmission electron microscopy (HR‐TEM) analysis was performed to further verify whether the charging oxides from the LMO and MnSO_4_ electrolytes were the same MnO_2_. Each sample was subjected to a voltage limit of 3.2 V after ten cycles. In the HR‐TEM image of the pristine MnO_2_ charged from MnSO_4_ (P‐MnO_2_), the d‐spacings of 2.56 Å and 1.56 Å corresponded to the (100) and (102) crystal planes of the ε‐phase, respectively (**Figure** **3**a).^[^ ^34^ ^]^ Moreover, the STEM‐EDS image detected Mn and O, supporting the formation of oxides through the Mn^2+^/MnO_2_ two‐electron redox reaction. The LMO electrolyte after the PR process showed the formation of the MnO_2_ (PR‐MnO_2_) after cycling (Figure 3b). The HR‐TEM results showed d‐spacings of 2.24 Å, 2.46 Å, and 1.64 Å, which were well matched to the (002), (100), and (102) crystal planes, respectively. These results implied that both P‐MnO_2_ and PR‐MnO_2_ had the same crystal structure. STEM‐EDS analysis of Mn and O also confirmed the formation of manganese oxide.

X‐ray photoelectron spectroscopy (XPS) measurements were conducted to observe the surface chemical characteristics of the charged products from MnSO_4_ and LMO electrolytes. The spectra from LMO powder (sky blue line), MnO_2_ charged from MnSO_4_ (P‐MnO_2_, black line), and MnO_2_ charged from the PR‐LMO (PR‐MnO_2_. red line) were calibrated to C 1s peak at 284.6 eV. The full scan of XPS surveys was recorded (Figure S10, Supporting Information). In the Mn 2p spectrum (Figure 3c), Mn 2p was assigned to Mn 2p_1/2_ and Mn 2p_3/2_, and each could be deconvoluted into two peaks, originating from Mn^3+^ and Mn^4+^.^[^ ^35^ ^]^ The Mn^4+^/(Mn^3+^ + Mn^4+^) ratio was ≈0.498, 0.525, and 0.347 for the LMO powder, P‐MnO_2_, and PR‐MnO_2_, respectively. The oxidation state of the LMO powder was 3.5, indicating an equal presence of Mn^3+^ and Mn^4+^. The PR‐MnO_2_ showed a higher proportion of Mn^4+^ ions.^[^ ^36^ ^]^ These results showed greater stability for a two‐electron transfer reaction. The O 1s peak was assigned to M‐O‐M (lattice oxygen), M‐O‐H (presence of oxygen vacancies), and adsorbed water around the surface (Figure 3d). The M‐O‐M peak in the LMO powder was attributed to bonding with Li metal. In the P‐MnO_2_, no M‐O‐M peak was observed. The O 1s XPS results confirmed that LMO underwent electrochemical reduction reactions and was deposited as MnO_2_. The M‐O‐H / ((M‐O‐M) + (M‐O‐H)) ratio increased significantly from 0.243 in the LMO powder to 0.772 in PR‐MnO_2_. The increased proportion of oxygen bonded to hydrogen (M‐O‐H) indicates the breaking of bonds between oxygen and lithium (Li‐O). This suggests that lithium ions were effectively removed during the reduction process, allowing Mn to form new oxidation states, which is consistent with the conversion of LMO to MnO_2_.

### 2.4 Optimization of the PR Process for Enhanced Mn Utilization and Stable Cell Performance

**Figure 4:**
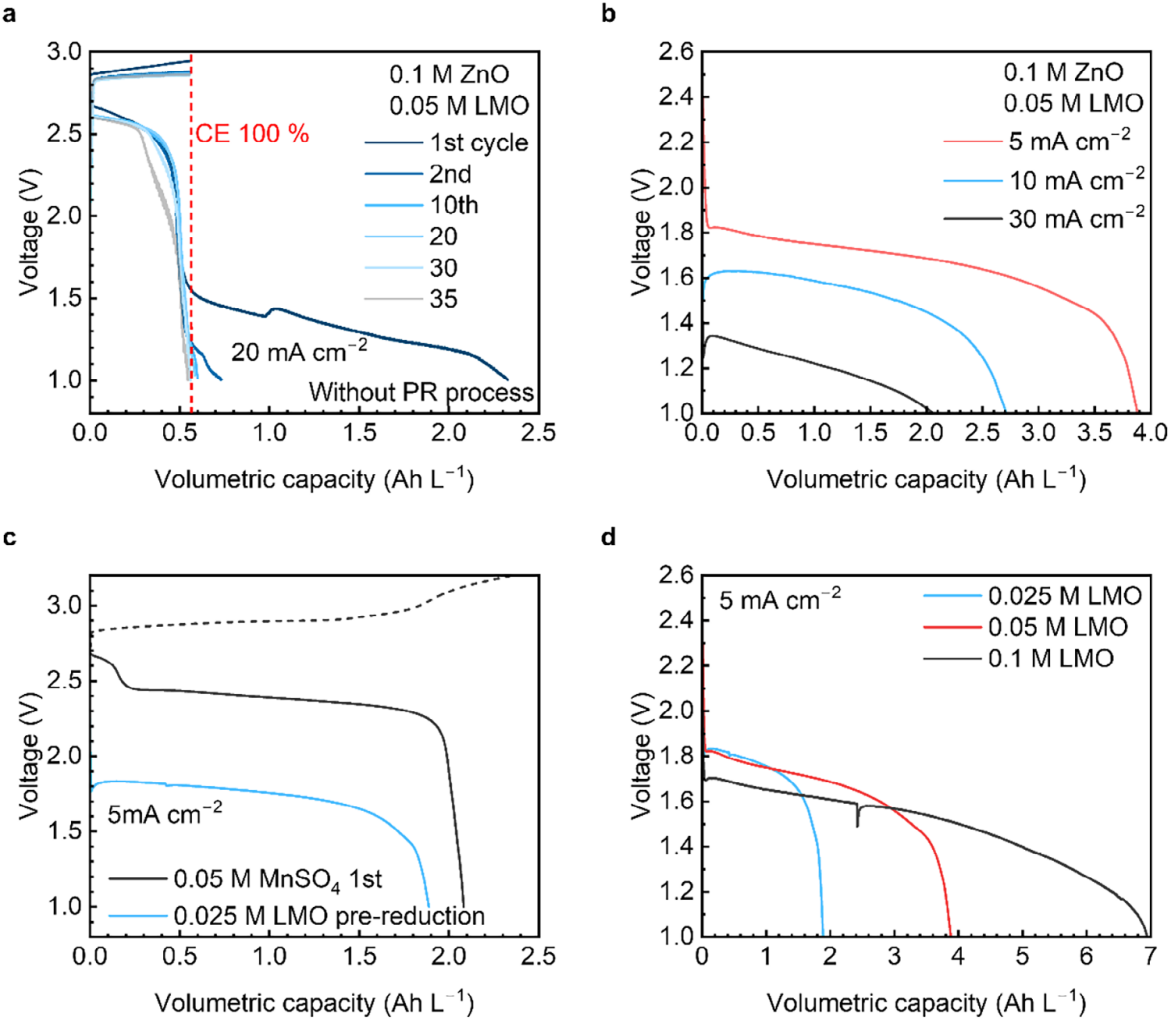
Galvanostatic charge‐discharge (GCD) profiles for the PR process optimization. All anolytes were composed of 0.1 m ZnO dissolved in 4 m NaOH solution. a) GCD profiles of the Zn‐LMO full cell until 35 cycles without PR. b) PR discharge profiles of the 0.05 m LMO catholyte at a current density of 5, 10, and 30 mA cm^−2^, respectively. c) GCD profiles of the full cell composed of 0.05 m MnSO_4_ at 20 mA cm^−2^ and 0.025 m LMO PR discharge profile. d) PR discharge profiles of the LMO catholyte with 0.025 m, 0.05 m, and 0.1 m LMO powder, respectively.

The PR process was optimized to efficiently utilize the Mn ions in the LMO and achieve stable cell performance. To compare with electrochemical reduction, we prepared an electrolyte with an additional reducing agent. Without the PR process, a solution chemically reduced by adding 4 wt.% H_2_O_2_, a strong reducing agent, to 2.5 m H_2_SO_4_ exhibited good charging performance (Figure S11, Supporting Information). However, the discharge retention rapidly decreased with repeated cycles, which was affected by the reducing agent. In **Figure** **4**a, the cell was tested by charging first, as in a conventional flow battery, with an electrolyte from the dispersed pristine‐LMO (P‐LMO). During the first charge cycle, Mn ions dissolved in sulfuric acid participated in the charging reaction, but a high overvoltage occurred, which was attributed to the insufficient amount of Mn ions. During the subsequent discharge process, two plateau regions appeared, which corresponded to the reduction of LMO observed in the PR stage. The difference in discharge voltages between the charged oxide (MnO_2_) and LMO confirmed the occurrence of specific reactions. In the following cycles, the discharge contribution from LMO gradually decreased. The coulombic efficiency dropped below 100% from the 30th cycle onward when the LMO reduction reaction was completed (Figure S12, Supporting Information).

The electrolyte composed of 0.050 m LMO for the PR process was optimized by changing the current density to 5, 10, and 30 mA cm^−2^, respectively. The voltage and capacity of PR reaction decreased as the current density increased (Figure 4b). The utilization rate of Mn was 92.6%, 64.2%, and 47.6% at 5, 10, and 30 mA cm^−2^, respectively. Accordingly, the current density was optimized at 5 mA cm^−2^. The discharge capacity was compared between the electrolytes from 0.050 m MnSO_4_ and LMO with an equiv. Mn ion concentration of 0.025 m (Figure 4c). The discharge capacity of the electrolyte from MnSO_4_ was obtained after charging to a cutoff of 3.2 V, showing a gap of 0.2 Ah L^−1^ in volumetric capacity. However, excluding the 0.2 Ah L^−1^ plateau at 2.7 V caused by side reactions during charging MnSO_4_ solution to 3.2 V, the electrolyte from the LMO showed the same discharge capacity of 1.9 Ah L^−1^, resulting in most of the 0.025 m LMO reacted.

The discharge capacity was evaluated at the same current density of 5 mA cm^−2^ and a CF area of 5 cm^2^, varying the LMO concentration (Figure 4d). LMO concentrations of 0.025 m and 0.050 m showed discharge capacities of ≈2.0 Ah L^−1^ and 4.0 Ah L^−1^, respectively, which were proportional to those of the MnSO_4_. However, at an increased concentration of 0.1 m, the discharge capacity was ≈7.0 Ah L^−1^, showing the discharge overvoltage caused by the high powder concentration with a low ion conductivity. The utilization rate of Mn, based on 0.05 m MnSO_4_, was 90.5%, 92.6%, and 83.3% for 0.025 m, 0.05 m, and 0.1 m LMO catholyte, respectively. Additionally, some LMO powder remained in the solution, not filtered properly because of the physical limitation of the lab‐scale flow cell. The catholyte capacity composed of the electrolyte after the PR process was equiv. to the first cycle discharge capacity. This indicates that the PR catholyte capacity determined the total cell capacity, demonstrating that the entire amount of filtered LMO available for reaction was fully utilized. (Figure S13, Supporting Information).

### 2.5 Electrochemical Kinetics and Ion Concentration Analysis of LMO and Conventional Electrolytes

**Figure 5:**
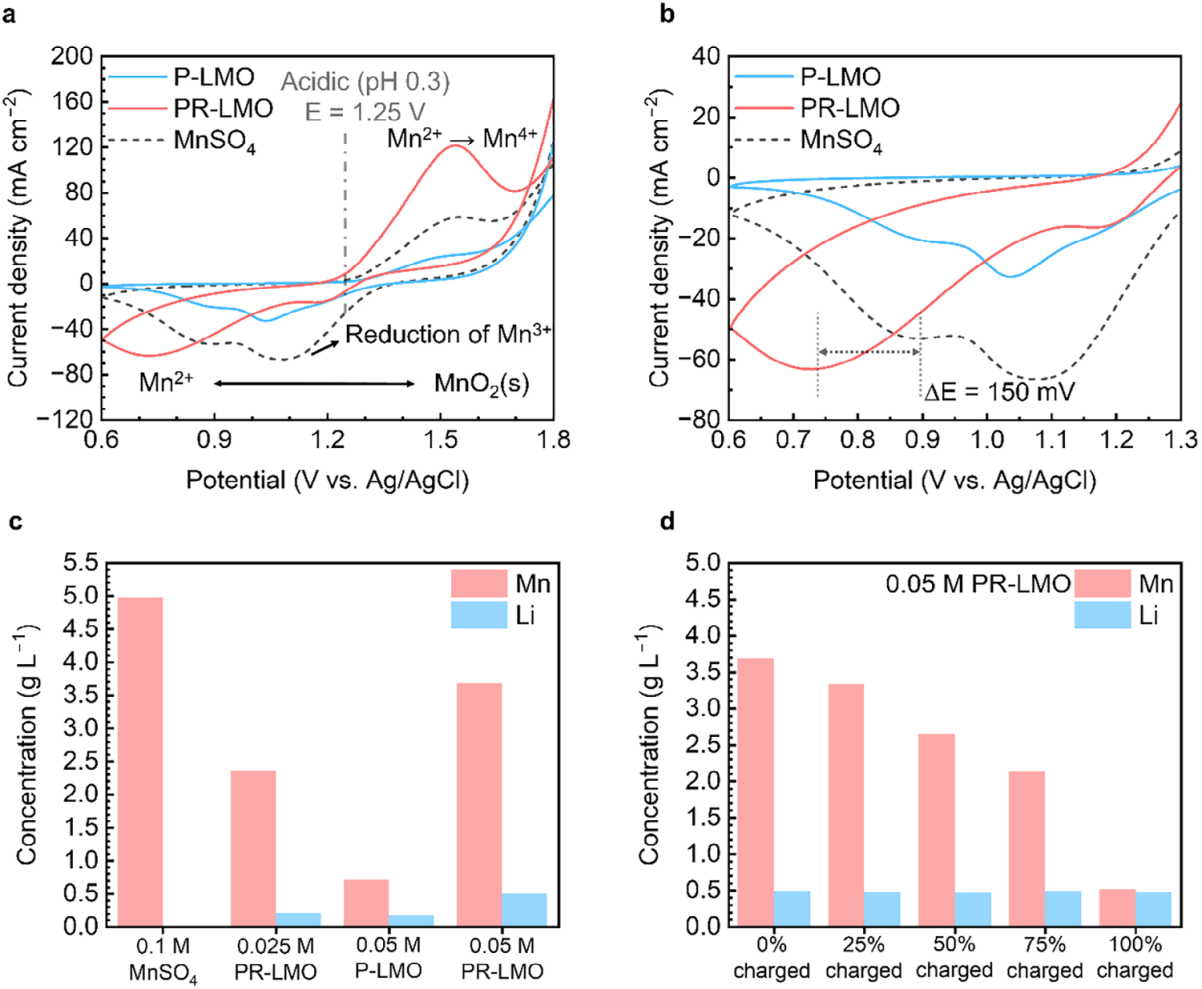
All samples were dissolved in 2.5 m sulfuric acid. a) Cyclic voltammetry curves (CV) of the electrolytes composed of pristine LMO, pre‐reduction LMO (PR‐LMO), and MnSO_4_ at a scan rate of 20 mV s^−2^, and b) disclosed curves. c) ICP‐OES analysis of the pristine 0.1 m MnSO_4_, 0.025 m PR‐LMO, 0.05 m P‐LMO, and 0.05 m PR‐LMO. d) ICP‐OES analysis of 0.05 m PR‐LMO catholyte at various states of the charging up to 100%.

The electrochemical kinetics of the electrolytes of MnSO_4_, pristine LMO, and PR‐LMO were characterized by cyclic voltammetry (CV). The MnSO_4_, pristine LMO, and PR‐LMO all showed the same oxidation peak at 1.55 V versus SCE (**Figure** **5**a). However, the pristine LMO that did not undergo the PR process, showed an oxidation peak of ≈20 mA cm^−2^, which was lower than the 120 mA cm^−2^ for the PR‐LMO at the same molar concentration. These results revealed that the concentration of Mn ions available for the reaction was lower in pristine LMO, implying that Mn ions were sufficiently reduced during the PR process to participate in oxidation reactions. There was a difference in the electrochemical reversibility during the reduction process between the pristine LMO and PR‐LMO compared to the reduction of conventional MnSO_4_ electrolyte. We observed a difference in the two‐electron reduction reaction from MnO_2_ to Mn^2+^. Additionally, we measured the full‐cell standard redox potential as 2.64 V based on the redox couples of Zn/[Zn(OH)_4_]2^−^ at −1.39 V versus Hg/HgO in the ZnO electrolyte and Mn^2+^/MnO_2_ at 1.25 V versus Ag/AgCl in the LMO electrolyte (Figure S14, Supporting Information). Both pristine LMO and MnSO_4_ showed a peak potential of 880 mV, whereas PR‐LMO exhibited a lower reversibility at 730 mV (Figure 5b). This difference was confirmed as a discharge peak shift of Mn ion that had undergone electrochemical reactions, as seen in the CV results before and after cycling of the MnSO_4_ (Figure S15, Supporting Information).

The ion concentrations of Mn and Li ions in the electrolyte from the pristine LMO and PR‐LMO were evaluated by inductively coupled plasma optical emission spectrometry (ICP‐OES) (Figure 5c). In 0.10 m of the MnSO_4_, the concentration of Mn ion was ≈5.0 g L^−1^, while 0.025 m of the PR‐LMO showed a concentration close to half at 2.4 g L^−1^, suggesting that most of the 0.05 m Mn had been reduced. The electrolytes of the pristine LMO and PR‐LMO were also evaluated at a concentration of 0.05 m LMO. For the electrolyte from pristine LMO, 0.7 g L^−1^ of Mn was detected, which increased to 3.7 g L^−1^ after the PR process. However, the Mn concentration of the 0.05 m PR‐LMO was 1.1 g L^−1^ less than the result for double that of 0.025 m PR‐LMO. This was interpreted as a decrease in overall discharge efficiency, which was related to the limited surface area of the CF in the lab‐scale flow cell.

Figure 5d shows the ICP results during the charging process up to a voltage limit of 3.2 V for 0.05 m PR‐LMO. The Mn concentration of 3.5 g L^−1^ decreased as the charging process progressed, and when charging ended at 3.2 V, the Mn ion concentration dropped to 0.5 g L^−1^. Also, the Li‐ion concentration remained constant at 0.5 g L^−1^ during and after the charging process, confirming that only Mn participated in the charging reaction.

### 2.6 Performance and Ion Recovery of Zn‐LMO Hybrid RFB with PR‐LMO Electrolyte

The spent LMO was obtained from the LIB pouch cells, which were cycled until a capacity retention of 80% (Figure S16, Supporting Information). After discharge to 3.0 V, the batteries were disassembled, and only the cathode was recovered. The cathode was processed according to the procedure presented in Figure 2a to obtain a powder containing the spent LMO and conductive agent (Figure S17, Supporting Information). To ensure comparison at the same molar concentration of Mn ions, 15 mL of catholyte was prepared by dissolving either 0.1 m MnSO_4_ or 0.05 m LMO in a 2.5 M H_2_SO_4_ solution. The 1.0 mL intermediate electrolyte of 3.0 m Na_2_SO₄ was injected between the membranes, and the anolyte consisted of 30 mL of 0.1 m ZnO dissolved in 4.0 m NaOH solution. The electrode was CF with a thickness of 4 mm with an area of 2 cm × 2.5 cm. The spent LMO active material underwent the PF process followed by the PR process at 5 mA cm^−2^ prior to cycling. Both charge and discharge processes were carried out at 20 mA cm^−2^. For the MnSO_4_ and PR‐LMO, the charging process was limited to 400 s with a maximum charging voltage of 3.2 V, and the discharge process was limited to 1.0 V.

**Figure 6:**
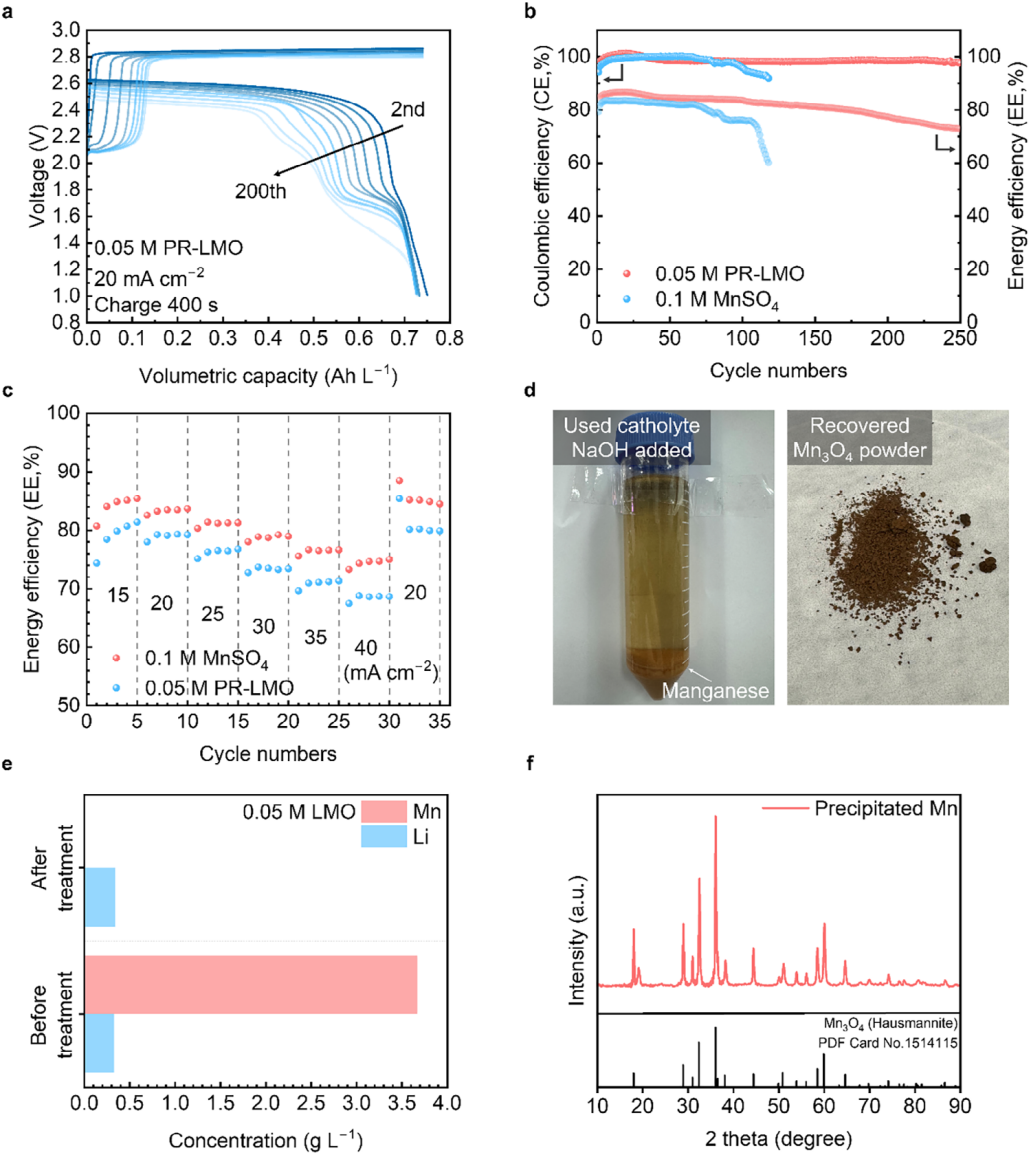
GCD profiles and efficiency in the Zn‐LMO hybrid RFB. a) GCD profiles of the Zn‐LMO hybrid RFB with 0.05 m PR‐LMO electrolyte at a current density of 20 mA cm^−2^ until 200 cycles and b) efficiency of the samples. c) EE while increasing the current density every five cycles and returning an initial current density of 20 mA cm^−2^. d) The image of the used catholyte after the 4 m NaOH added and recovered Mn_3_O_4_ powder after vacuum filtration. e) ICP‐OES analysis of 0.05 m used LMO catholyte before and after the pH treatment. f) XRD surveys of the precipitated Mn (hausmannite) with PDF Card (1514115).

The Zn‐LMO hybrid RFB test composed of a zinc anolyte and the PR‐LMO electrolyte was evaluated under constant current conditions. **Figure** **6**a shows the charge‐discharge profiles of the Zn‐LMO hybrid RFB containing the PR‐LMO electrolyte until 200 cycles. Consistent with the previous CV results, the Mn^2+^/Mn^3^⁺ reaction led to a gradual decrease in EE as the cycles progressed. The hybrid RFB with conventional MnSO_4_ electrolyte also exhibited a similar charge‐discharge plateau (Figure S18, Supporting Information).

Figure 6b shows the coulombic efficiency and EE of the full cell. The Zn‐LMO hybrid RFB with PR‐LMO electrolyte showed coulombic efficiency exceeding 95% and EE above 70% during 250 cycles. Furthermore, the discharge capacity was greatly maintained up to 250 cycles (Figure S19, Supporting Information). The high‐concentration Zn‐LMO hybrid RFB test results showed no improvement in discharge capacity from the PR process, but CE of 95.1% and EE of 73.1% were maintained until 100 cycles. It was implied that the PR process at high concentration was limited because of the restricted surface area of the CF (Figures S20 and S21, Supporting Information).

Figure 6c shows the performance of the hybrid RFB at various current densities, with tests conducted over five cycles for each current density change. The MnSO_4_ and PR‐LMO showed EE values of 81.4% and 85.4%, respectively, at a current density of 15 mA cm^−2^. Remarkably, hybrid RFB with the PR‐LMO maintained an EE of 75.0% even at 40 mA cm^−2^. This result suggests that the Li‐ion reduced from the spent LMO in the electrolyte, along with H_2_SO_4_, contributed as the common ion effect, leading to enhanced kinetics even at high current densities.^[^ ^37^ ^]^ Although there was no significant difference in the series resistance (R_s_) values between the electrolytes with and without Li ions, a difference in charge transfer resistance was observed when considering the internal resistance of the cell (Figure S22, Supporting Information).

We separated the Mn and Li ions from the upcycled positive electrolyte of Zn‐LMO hybrid RFB for the reuse by simply adjusting the pH. In the spent electrolyte, both Mn and Li ions were dissolved in an ionic state. Figure 6d shows the image of Mn_3_O_4_ powder after adding 4 m NaOH solution to the positive electrolyte at a volume ratio of 1:2 and filtered the precipitate under pressure. When the acidic LMO used electrolyte was adjusted to an alkaline pH, Mn was combined with OH groups and eventually formed oxides (Equations 3 and 4).^[^ ^38^, ^39^ ^]^

The ICP results before and after adding NaOH showed that Mn ions were completely removed from the spent electrolyte of Zn‐LMO hybrid RFB (Figure 6e). In contrast, the Li ions remained unchanged and were unaffected by the pH change. Li ions in the solution have been extensively studied for use as a precursor for various cathode materials, and it is expected to be applicable. Figure 6f shows the x‐ray diffraction (XRD) results of the precipitated powder. The results support that Mn(OH)_2_ present in the solution was oxidized to Mn_3_O_4_.

(1) $$\mathrm{LiM}n_{2}O_{4}+8H^{+}+2e^{-}\to Li^{+}+2Mn^{2+}+4H_{2}O$$

(2) $$Mn^{2+}+2H_{2}O\to \mathrm{Mn}O_{2}+4H^{+}+2e^{-}$$

(3) $$Mn^{2+}\left(\mathrm{aq}\right)+2OH^{-}\left(\mathrm{aq}\right)\to \mathrm{Mn}{\left(\mathrm{OH}\right)}_{2}\left(s\right)$$

(4) $$6\mathrm{Mn}{\left(\mathrm{OH}\right)}_{2}\left(s\right)+O_{2}\left(g\right)\to 2Mn_{3}O_{4}\left(s\right)+6H_{2}O\;\left(l\right)$$

## 3 Conclusion

We reported the novel upcycling process of spent LMO from LIB pouch cells by directly integrating it into an aqueous Zn‐LMO hybrid RFB system. By analyzing the electrochemical performance and charge‐discharge characteristics of the spent LMO electrolyte compared to the conventional MnSO_4_ electrolytes, it was demonstrated that the spent LMO electrolyte could be effectively used in the hybrid RFB system. Also, after sufficient reduction of Mn ions through PR process of the LMO electrolyte, it could be used for charge‐discharge cycles. The Zn‐LMO hybrid RFB system showed excellent cycle stability over more than 200 cycles, which represented about twice the cycle performance compared to the conventional electrolyte. We observed the good electrochemical stability under various current density conditions, particularly maintaining an EE of over 75% even at 40 mA cm^−2^, exhibiting excellent performance under high current. Furthermore, the possibility of Mn and Li ion recovery process was demonstrated through a simple process in which NaOH was used to increase the pH of the used electrolyte and separate Mn ions as Mn_3_O_4_ precipitate. The Li ions remained stable in the electrolyte during the process.

In conclusion, we proposed the great potential to directly apply the spent LMO to the hybrid RFB systems, thereby addressing the challenges of LIB recycling while enhancing the economic feasibility and sustainability of energy storage devices. Future studies should include experiments on recycling other cathode materials containing valuable metals, such as LiFePO_4_ (LFP), LCO, NCM811, and NCM622 through hybrid RFBs, with the development of suitable electrolytes. This will require the exploration of iron or cobalt‐based RFBs and an in‐depth investigation of the redox potentials of each metal. In addition, it will be promising to explore specific methods for utilizing Li ions within the electrolyte. We believe that the direct integration of spent LIB cathode materials with hybrid flow batteries opens a new horizon in the field of recycling and greatly contributes to achieving a circular economy.

## 4 Experimental Section

### Materials

The following chemicals and materials are all commercially available and were used as received: manganese sulfate monohydrate, sulfuric acid, zinc oxide, sodium hydroxide, zinc metal, and lithium sulfate monohydrate (all from Sigma Aldrich), cation exchange membrane (Nafion 117, Full Cell store, USA), anion exchange membrane (Selemion DSV, Asahi glass, Japan), CF (4 mm thick, XF30A activated CF, TOYOBO, Japan), lithium manganese oxide(MTI Corporation, USA), artificial anode (s360, Noticeable Battery Total Solution(NBTS), South Korea), copper foil (TPC C1100, 15 um, UACJ, Japan), aluminum foil (20 um, NBTS), separator (2400, Celgard, USA), pouch tab (Aluminum, Nickel, 0.1 T, 2 W, NBTS). 1 m lithium hexafluorophosphate (LiPF_6_) in ethylene carbonate: diethyl carbonate = 1:1 (Soulbrain, South Korea) was used for the pouch cell electrolyte. All aqueous electrolytes were manufactured using deionized water by an ultrapure water production system (18.2 mΩ cm, HIQ).

### LMO Pouch Full Cell Fabrication

The pouch full cell used to obtain spent electrodes was fabricated as a single‐layer cell. The cathode consisted of 92 wt.% active material (LiMn_2_O_4_), 4 wt.% conductive agents (Super P), and 4 wt.% binders (polyvinylidene fluoride, PVDF) dissolved in anhydrous N‐methyl‐2‐pyrrolidone (NMP). The mixture was processed using a Thinky Mixer (ARE‐310, THINKY USA) at 2000 rpm for 20 min, followed by degassing at 2 200 rpm for 30 s. The slurry was coated onto aluminum foil using a coating machine (Rohtec, South Korea) and a doctor blade (Mitutoyo). The coated cathode was dried at 80 °C for 12 h. The anode consisted of 92 wt.% artificial graphite, 4 wt.% conductive agent (Super P), and 4 wt.% binder (SBR dissolved in water: carboxymethyl cellulose = 1:1 by weight). Mixing and coating were carried out under the same conditions as for the cathode. The coated anode was dried in a vacuum oven at 60 °C for 12 h. The dried electrodes were roll‐pressed to achieve a loading density of 2.0 g cm^−3^ for the cathode and 1.6 g cm^−3^ for the anode. The electrodes were laser cut (LSU3 EB, HGTECH) to a size of 12 cm^2^ based on the cathode dimensions and dried in a vacuum oven at 60 °C for 12 h. For the fabrication of the pouch cell, an aluminum tab was ultrasonically welded to the cathode, and a nickel tab was welded to the anode. The anode, separator, and cathode were fixed in place with polyimide tape, and an initial sealing was performed. Subsequently, 1 mL of 1 m lithium hexafluorophosphatein ethylene carbonate (1:1 by weight) was injected. A second vacuum sealing was performed, followed by aging for 24 h to allow electrolyte impregnation. The cell was assembled in a dry room. The LMO full cell was charged and discharged within a voltage range of 3.0 to 4.3 V. After formation at 0.2 C for three cycles, cycling was conducted at 0.5 C. The cell lifespan was defined as the number of cycles until 80% degradation of the initial capacity.

### Preparation of the Catholyte with Spent LMO

The spent LMO pouch cell was disassembled by cutting off the tabs and seals, and only the cathode was separated. The cathode material was scraped off and separated from the current collector, then ground using a mortar. The ground powder was placed in NMP solvent at a ratio of 10 mL per gram of powder and heated at 80 °C for 6 h with stirring to remove PVDF from the powder. The powder was then separated from the NMP solvent using a centrifuge at 8000 rpm for 10 min, followed by another centrifugation with deionized water at 8000 rpm for 10 min. The powder, free of salts, was dried in a vacuum oven at 60 °C for 6 h. The obtained powder consisted of LMO and Super P in a ratio of 92:4. A mixture of 0.05 m LMO and Super P was added to 2.5 m sulfuric acid solvent and dispersed using a tube mixer for 30 s.

### Manganese Cathode Redox Reaction Characterization

The PF, PR, and charged surfaces of the CF cathode were observed using field emission scanning electron microscopy (FE‐SEM, S‐4700, Hitachi and GEMINI 500, ZEISS). The oxides after charging were examined with a high‐resolution transmission electron microscope (HR‐TEM, TALOS F200X) operating at 200 kV. Elemental mapping of each sample was obtained by energy dispersive X‐ray spectroscopy (EDX) included in the FE‐SEM and HR‐TEM. The chemical bonding between Mn and O in the oxides was analyzed using XPS (AXIS Supra, Kratos Analytical Ltd.) with a monochromatic Al‐Kα source (1486.6 eV). The metal ion concentrations in the electrolyte were determined using inductively coupled plasma optical emission spectroscopy (ICP‐OES, Optima 8300, PerkinElmer). CV measurements were carried out with a three‐electrode system consisting of a 3 mm diameter platinum wire as the working electrode, CF (7 mm × 7 mm × 4 mm), a graphite counter electrode, and Ag/AgCl reference electrode in acidic conditions. The PR and post‐reduction of LMO, as well as MnSO_4_ electrolyte, were each prepared in 10 mL samples and measured using a potentiostat with a scan rate of 20 mV s^−1^.

### Aqueous Zinc‐LMO Hybrid Redox Flow Batteries from Spent LMO

The RFB used an anolyte of 30 mL of [Zn(OH)_4_]^2−^ prepared by dissolving 0.1 m ZnO in 4.0 m NaOH solution, and a catholyte consisting of either 0.05 m LMO powder dispersed or 0.1 m MnSO_4_ dissolved in 2.5 m H_2_SO_4_ solution. An anion exchange membrane (Selemion DSV) was used at the cathode, while a cation exchange membrane (Nafion 117) was used at the anode, with 1 mL of 3.0 m Li_2_SO_4_ as the middle electrolyte between the membranes. Each ion exchange membrane was secured in a 1 mm thick Teflon gasket, and 1 mL of the middle electrolyte was injected through a syringe needle. CF electrodes with an area of 2 cm × 2.5 cm and a thickness of 4 mm were used, with a bipolar plate and a copper plate positioned as the current collectors. The electrolyte flow rate was maintained at 25 mL min^−1^ for both PF and charge‐discharge cycling, using a peristaltic pump drive (JWSE100). The GCD profile was measured using a WBCS3000M1 (Wonatech.A), with current densities ranging from 5 mA cm^−2^ (PR) up to 40 mA cm^−2^. XRD (SmartLab, Rigaku Corporation) was used to determine the crystal structure of the deposited manganese oxide. Potentiostatic electrochemical impedance spectroscopy (PEIS) measurements were performed as required, covering a frequency range from 10 kHz to 0.5 Hz.

## Conflict of Interest

The authors declare no conflict of interest.

## Supporting information



Supporting Information

## Data Availability

The data that support the findings of this study are available from the corresponding author upon reasonable request.
