# Supplementary material for: Direct Integration of Spent LiMn2O4 with High Voltage Aqueous Zinc‐Manganese Redox Flow Batteries as a Practical Upcycling Process
Source: Small. 2025 Mar 11;21(32):2500787. doi: 10.1002/smll.202500787 (PMC12366278; doi:10.1002/smll.202500787)
Supplement: Supplementary file 1 — Supporting Information [file SMLL-21-2500787-s001.docx]

Supporting Information

Direct integration of spent LiMn_2_O_4_ with high voltage aqueous zinc-manganese redox flow batteries as a practical upcycling process

Duho Han, Hyeokjun Jang, Jinyeong Choi, Jihan Park, Seongmin Kim, Seongsoo Han, Wonjae Lee, Jin Hong Lee, Pilgun Oh*, Yosep Han*, Minjoon Park*

**
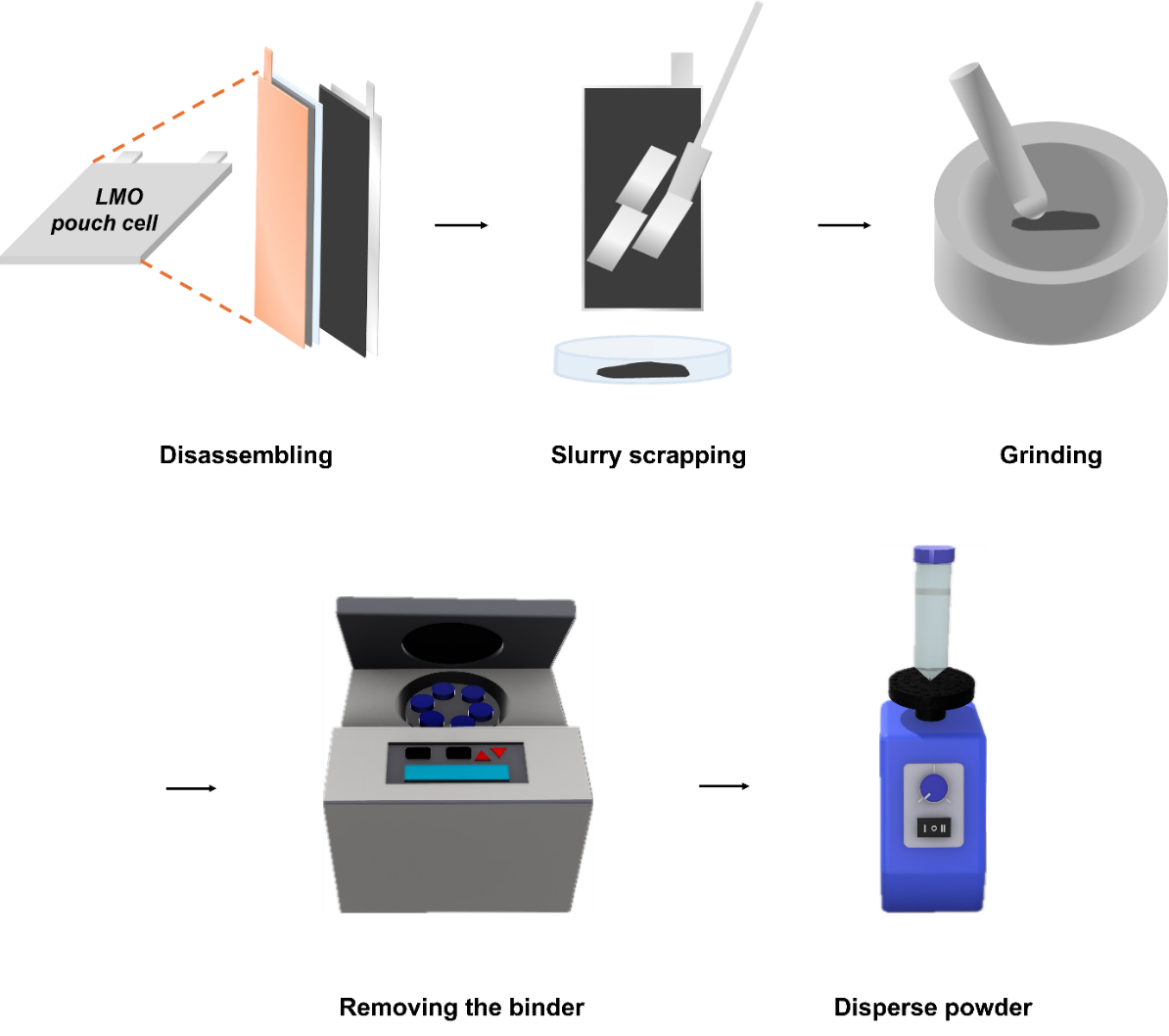
**

**Figure S1.** The schematic diagram illustrating the process of obtaining LMO catholyte.


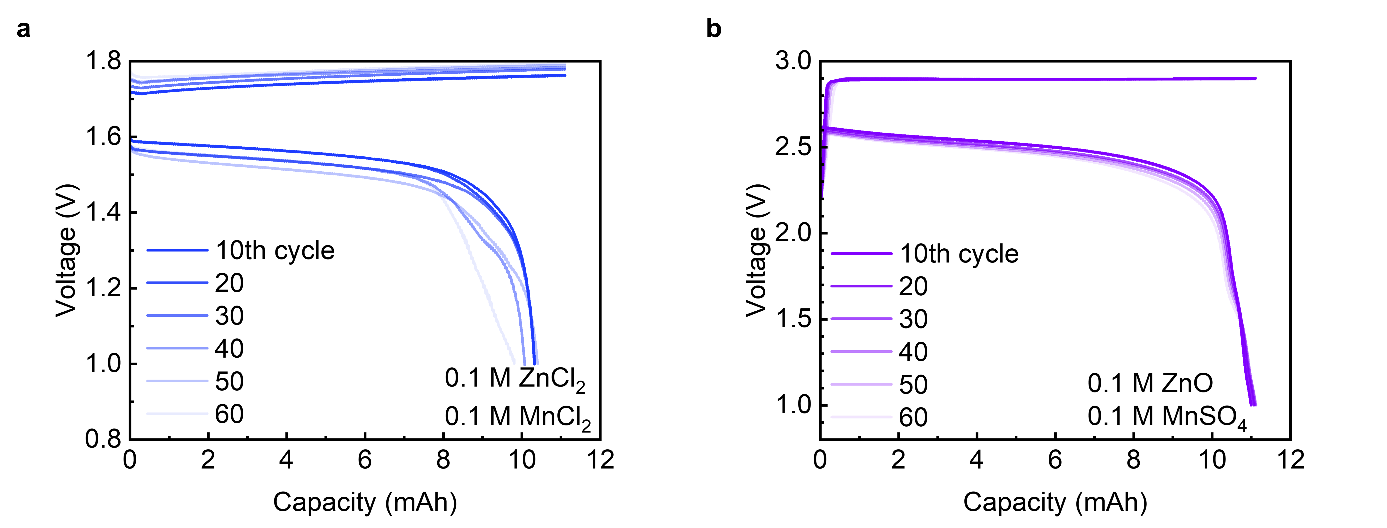


**Figure S2.** The galvanostatic charge-discharge profiles of the single membrane neutral system and double membrane hybrid system at a current density of 20 mA cm^−1^. (a) In the neutral system, a cation exchange membrane (Nafion117) and a 2.5 M NaCl supporting electrolyte were used as the supporting electrolyte. (b) In the hybrid system, two exchange membranes (Nafion 117 and Selemion DSV) were employed, with 4 M NaOH and 2.5 M H_2_SO_4_ as the supporting electrolyte. The pH difference between the electrolytes generated a voltage difference in the same zinc-manganese RFB system.


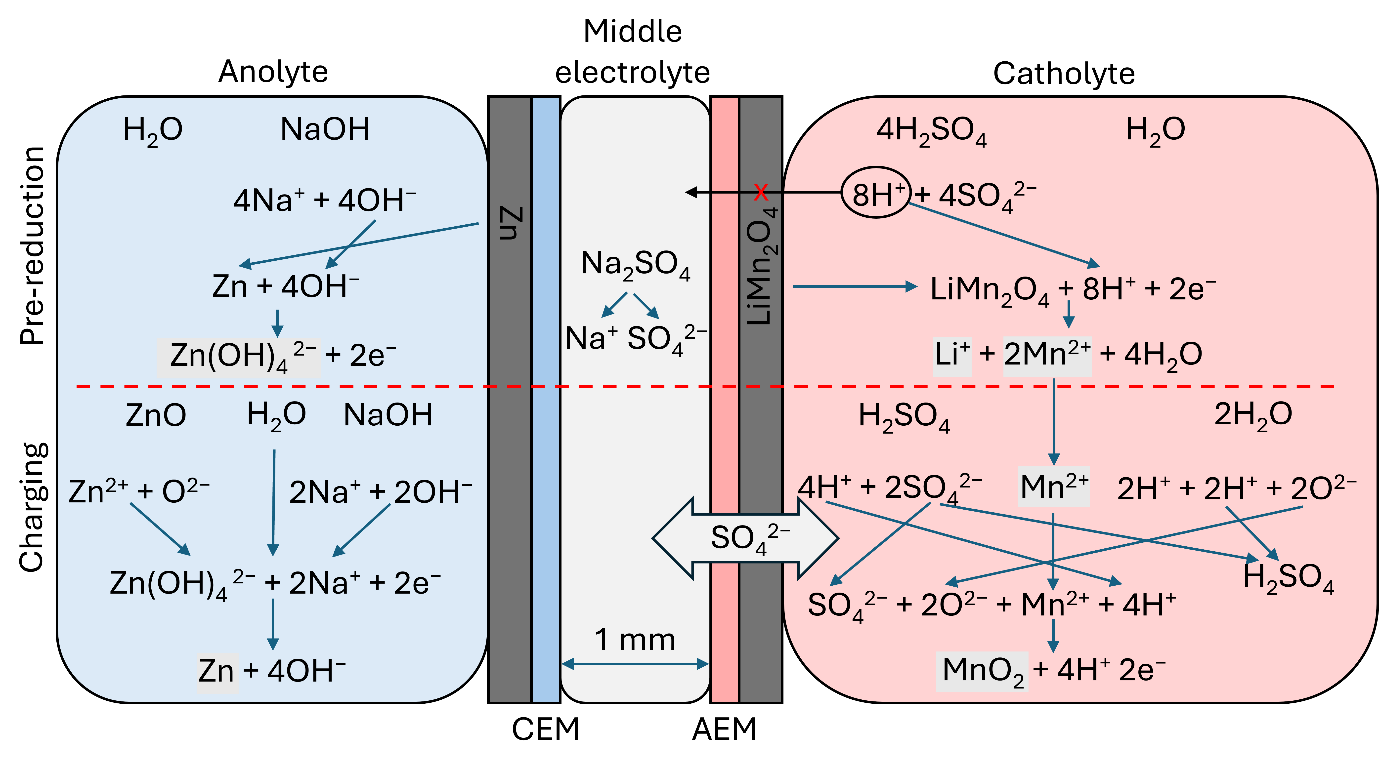


**Figure S3.** The pre-reduction and charging mechanisms of the upcycled hybrid zinc-manganese redox flow battery are depicted. The 1 mm width middle electrolyte layer and two ion exchange membranes effectively prevented the crossover of hydrogen ions (H^+^) and hydroxide ions (OH^−^), thereby maintaining the pH stability of the respective electrolytes. Zn metal is oxidized to soluble zincate (Zn(OH)_4_^2−^), while LMO is reduced to lithium (Li) and manganese (Mn) during the pre-reduction process. During the charging process, zincate at the anode is reduced to Zn metal, and Mn at the cathode is oxidized to MnO₂, with Li remaining non participatory in the reaction.


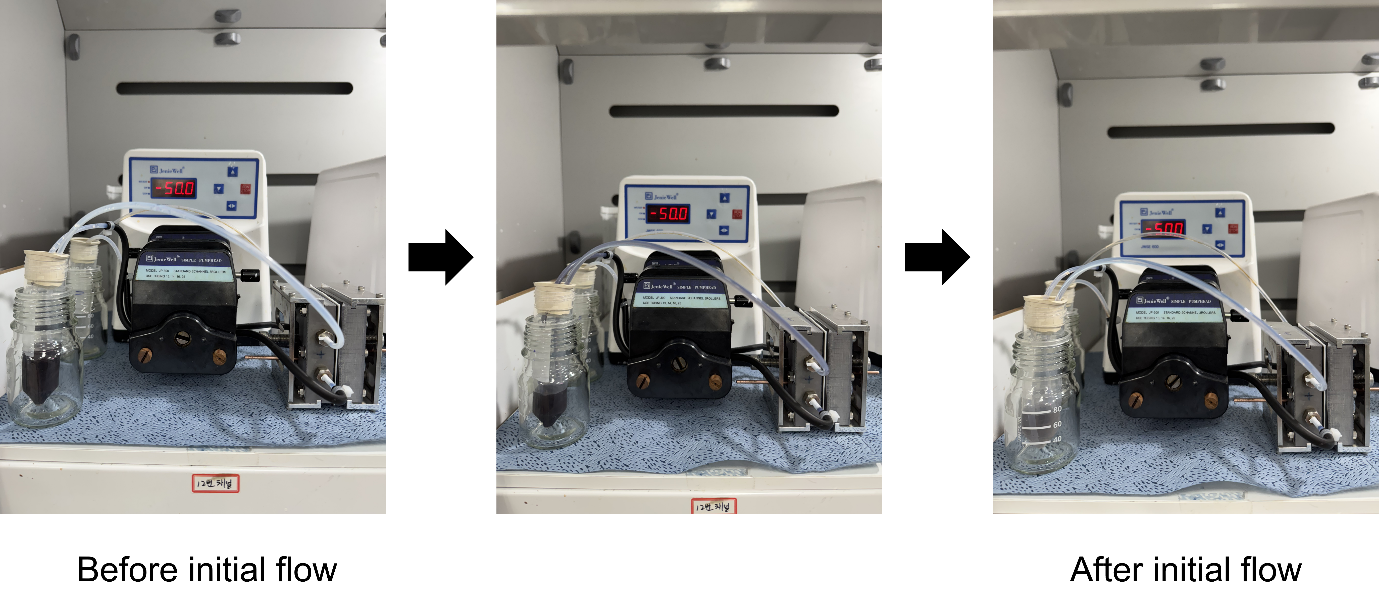


**Figure S4.** Images of the RFB stack before and after pre-filtration, without the current applied.


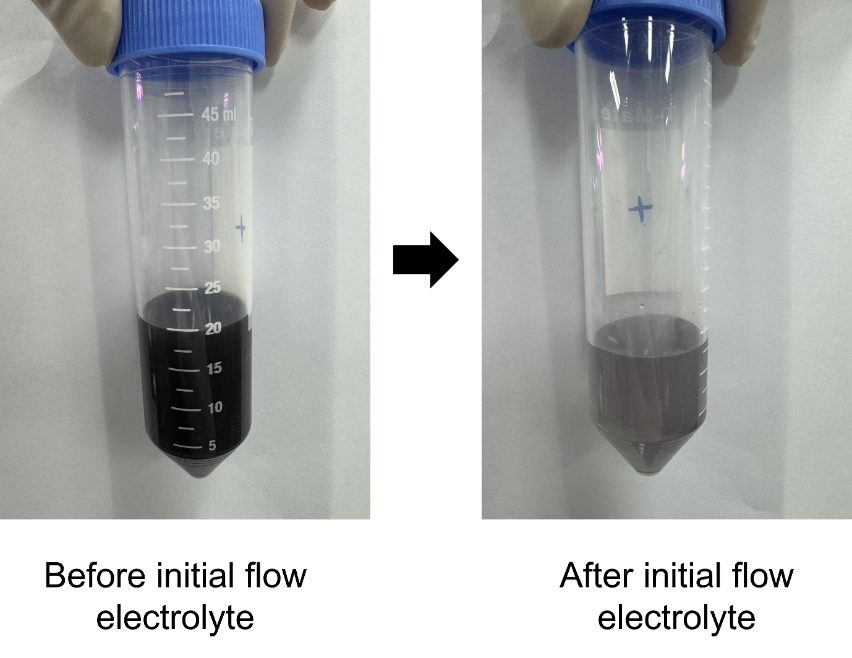


**Figure S5.** Images before and after the initial flow. The LMO dispersed in the electrolyte became transparent after the flow.


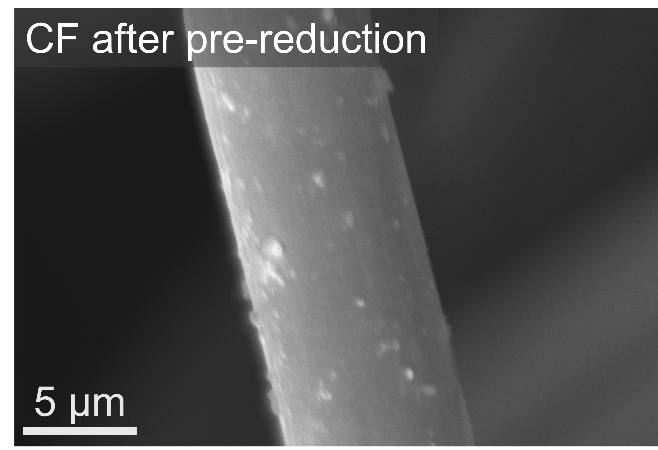

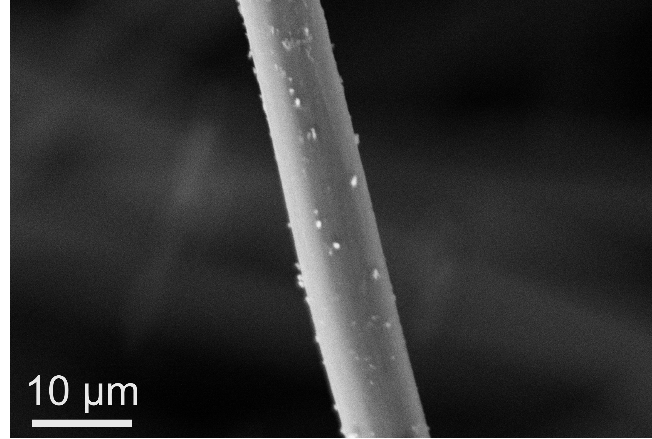


**Figure S6.** The SEM images of CF after pre-reduction process.


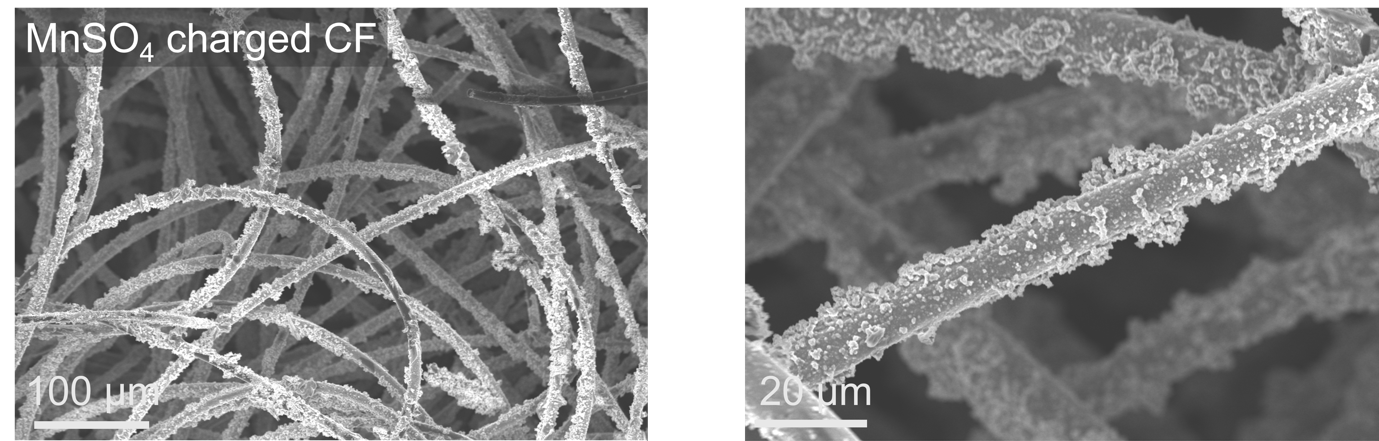


**Figure S7.** The SEM images of CF charged from the MnSO_4_ electrolyte.


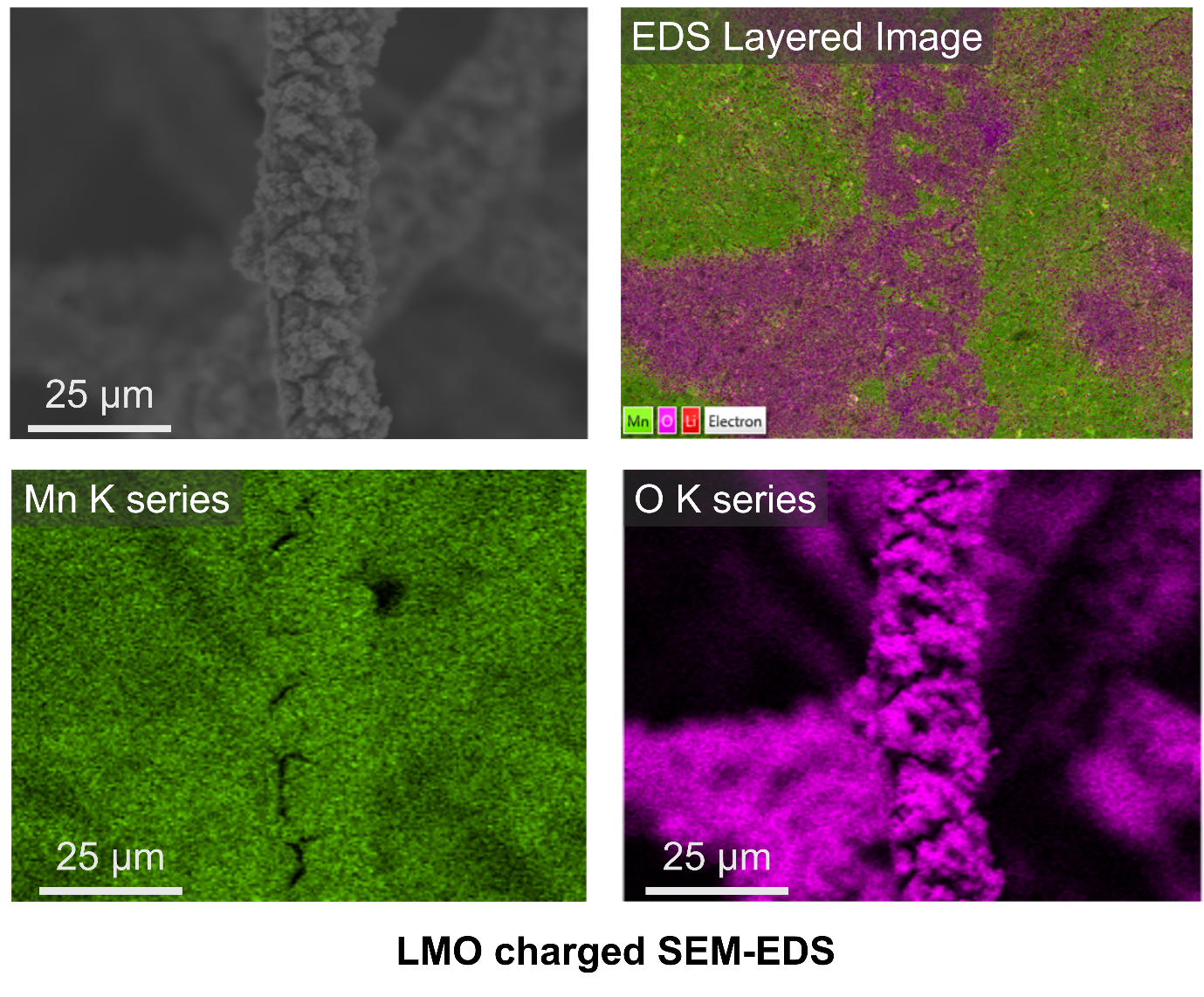


**Figure S8.** SEM-EDS analysis of the CF charged from the LMO catholyte, showing manganese and oxygen elements. Scale bars, 25 um.


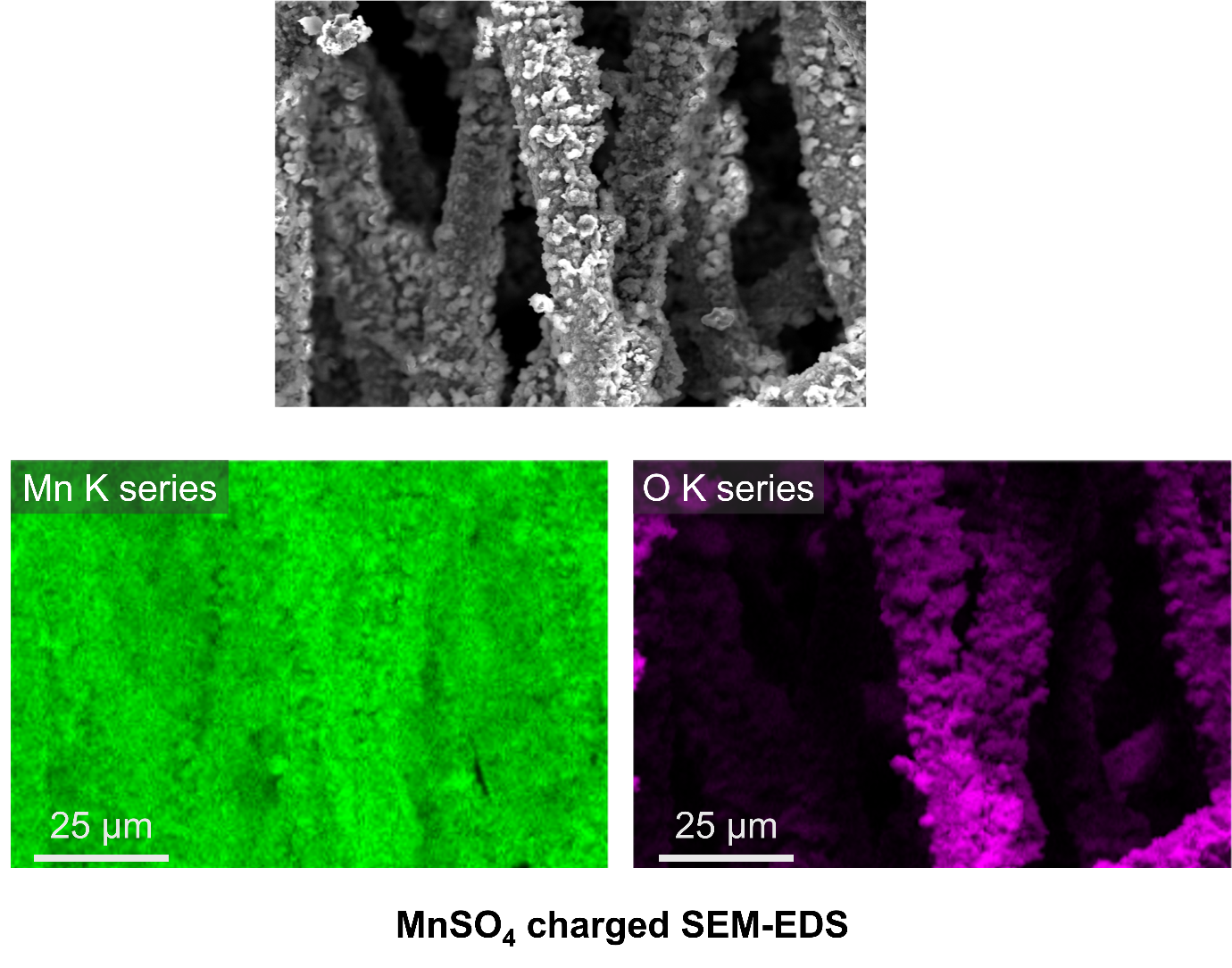


**Figure S9.** SEM-EDS analysis of the CF charged from the MnSO_4_ catholyte, showing manganese and oxygen elements. Scale bars, 25 um.


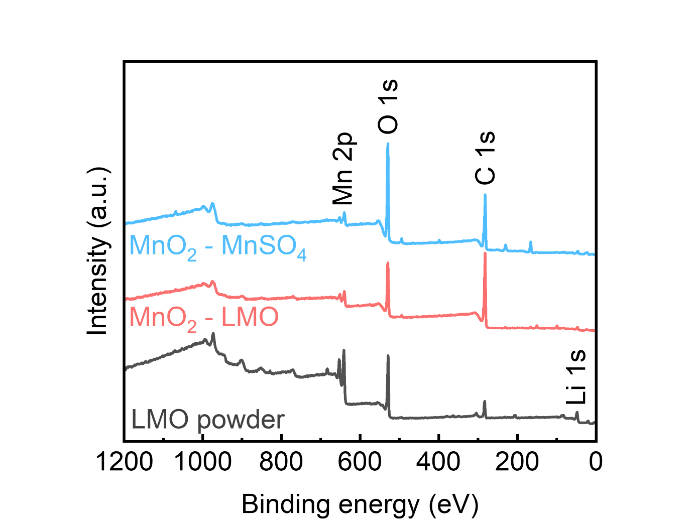


**Figure S10.** The full scan surveys of the LMO powder, MnO_2_-LMO, and MnO_2_-MnSO_4_.


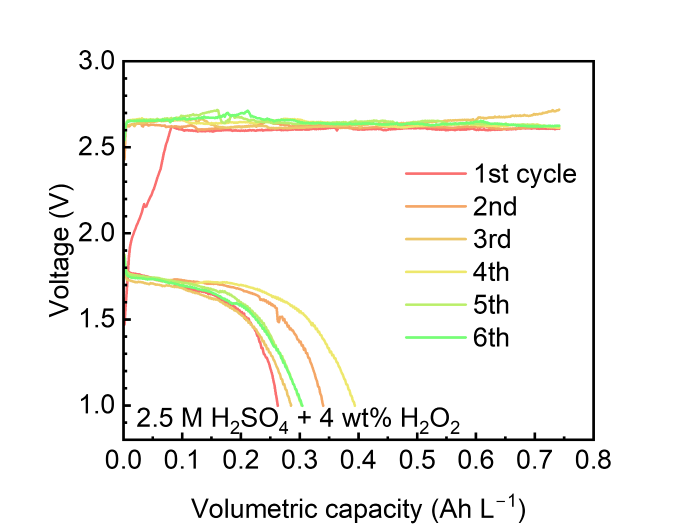


**Figure S11.** Galvanostatic charge-discharge profiles of the Zn-Mn RFB with 4 wt% H_2_O_2_ added to the MnSO_4_ catholyte


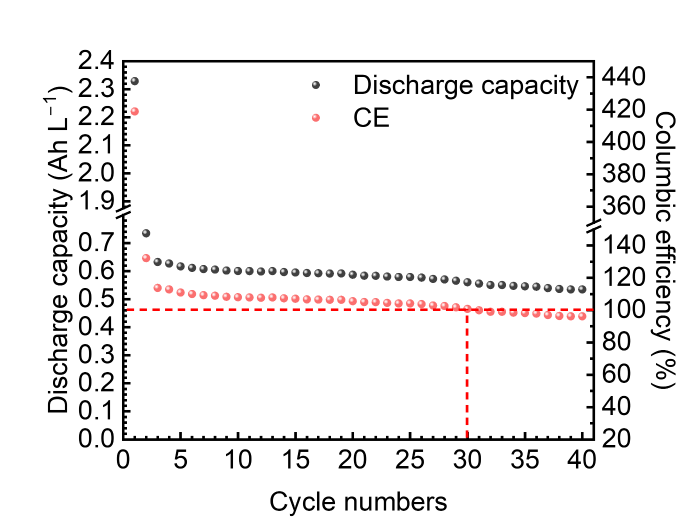


**Figure S12.** Discharge capacity and coulombic efficiency of the cell without pre-reduction process up to 40 cycles.


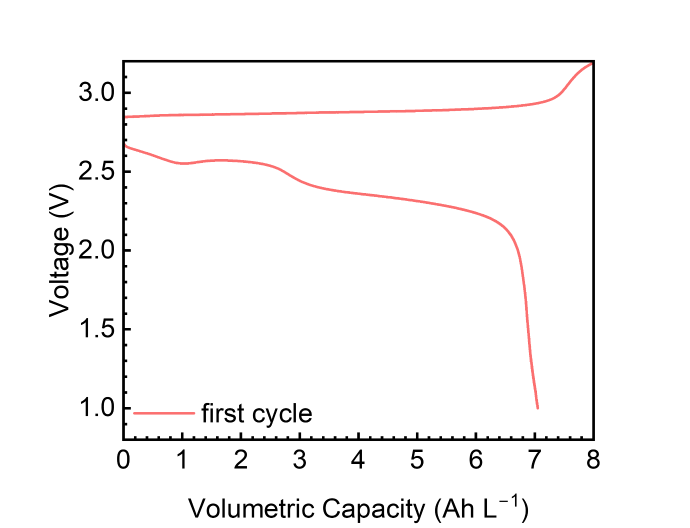


**Figure S13**. Galvanostatic charge-discharge profile of the first cycle after the pre-reduction at 5 mA cm^−2^ for 0.1 M LMO.


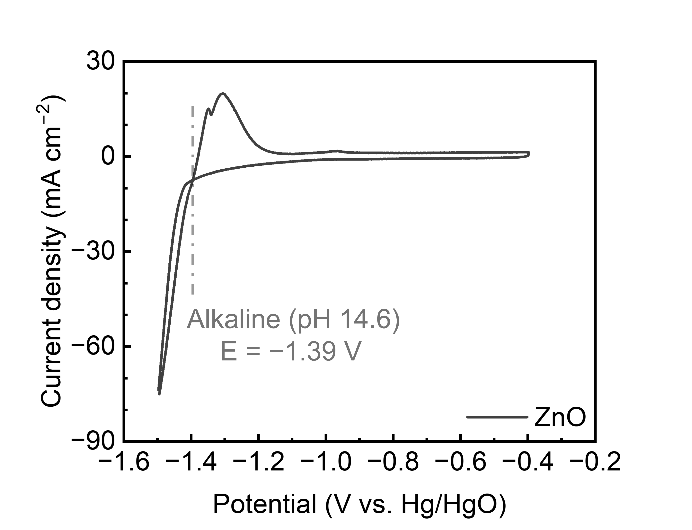


**Figure S14**. Cyclic voltammetry curves of the 0.1 M ZnO electrolyte at scan rate 20 mV s^−1^.


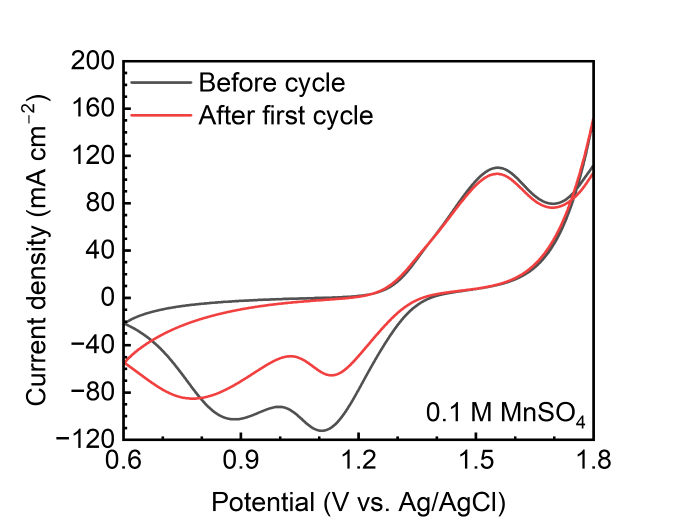


**Figure S15.** Cyclic voltammetry curves of the 0.1 M MnSO_4_ electrolyte before cycle and after cycle at scan rate 20 mV s^−1^.


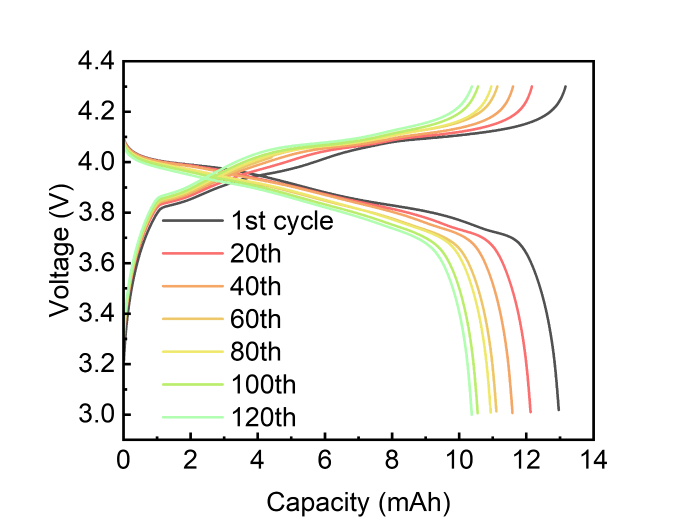


**Figure S16.** The galvanostatic charge-discharge profiles of LMO pouch full cell up to 80% of their initial capacity.


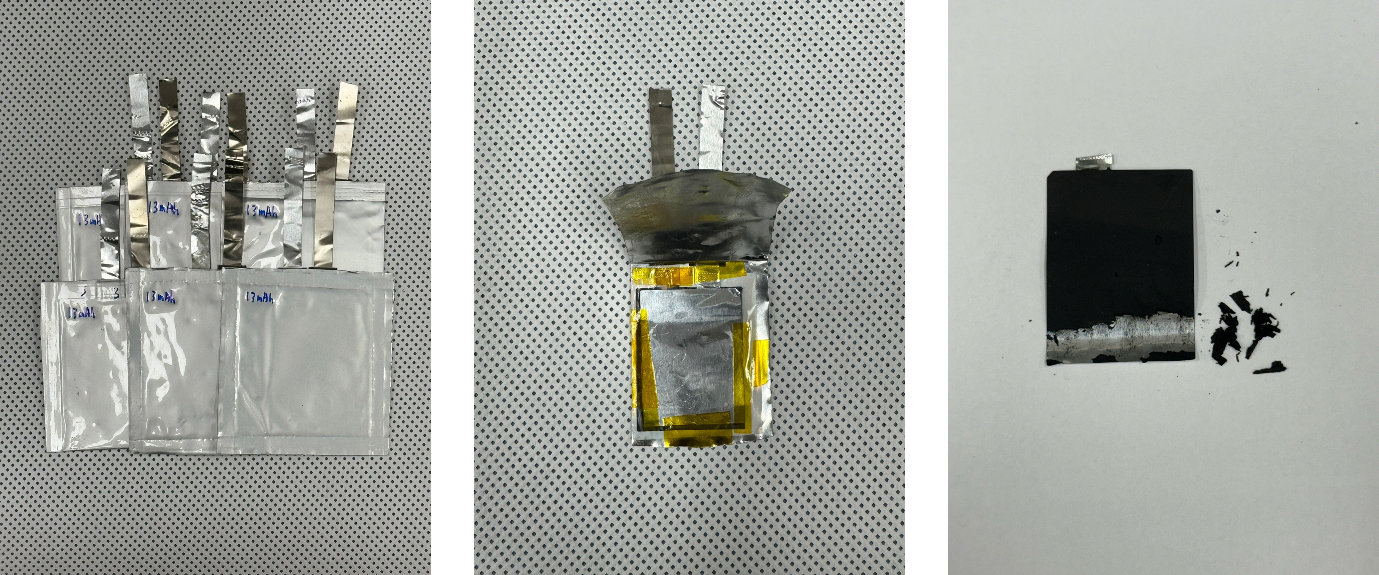


**Figure S17.** Photos of the disassembled LMO pouch cells, showing the process of obtaining the used cathode material.


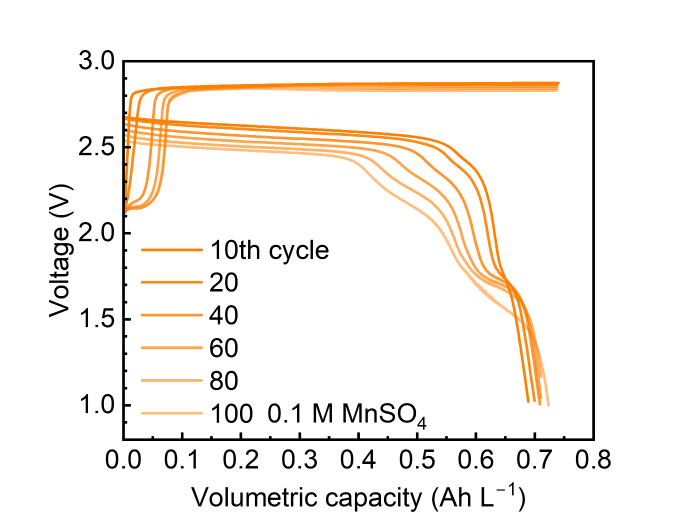


**Figure S18.** Full cell charge-discharge profiles with the catholytes composed of 0.1 M MnSO_4_.


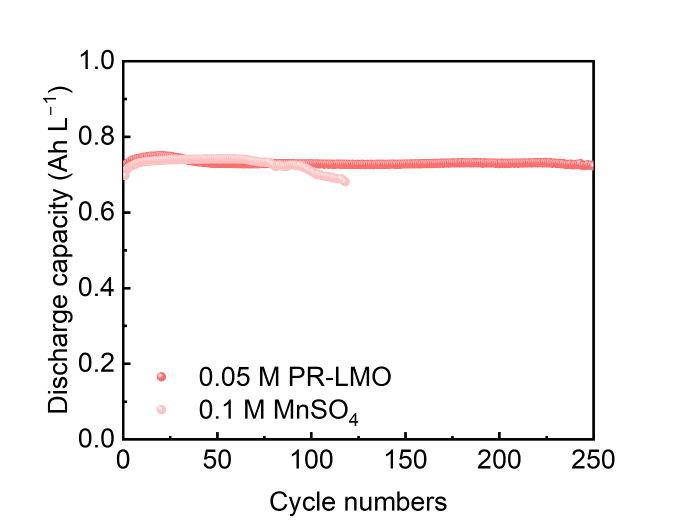


**Figure S19.** The discharge capacity of Zn-Mn hybrid full cell with 0.05 M PR-LMO and 0.1 M MnSO_4_, respectively.


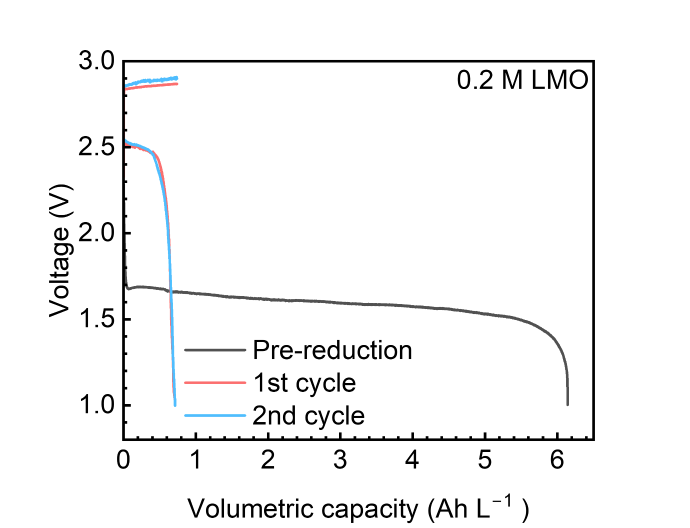


**Figure S20.** The galvanostatic charge-discharge profiles of high-concentration 0.2 M LMO for the pre-reduction and up to 2nd cycle.


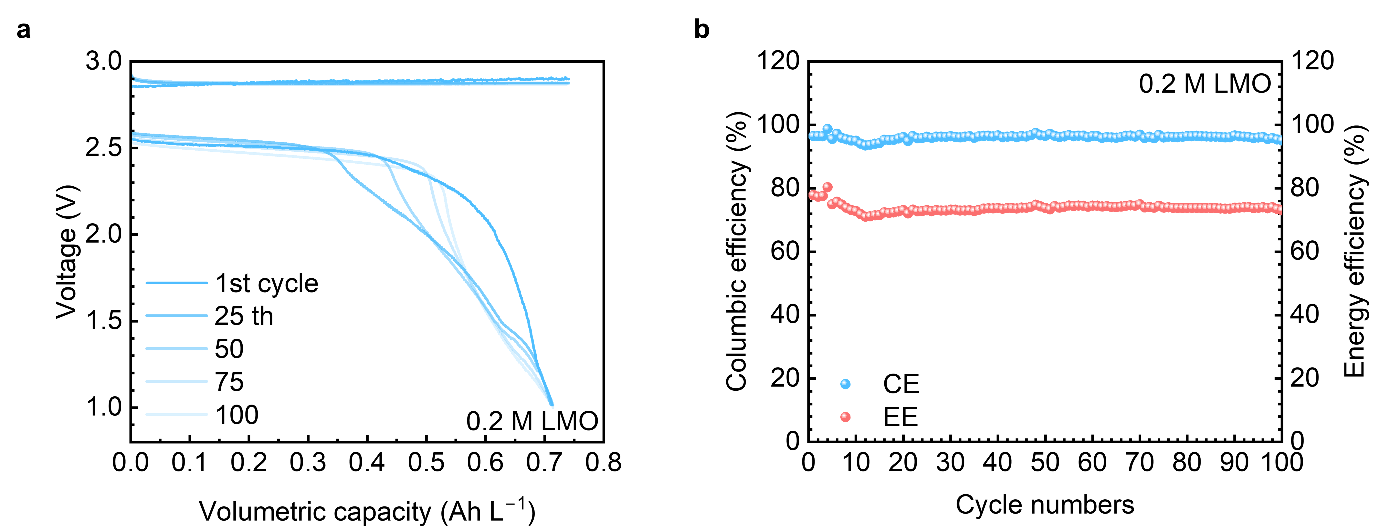


**Figure S21.** (a) The galvanostatic charge-discharge profile of high-concentration 0.2 M LMO up to 100 cycles, and (b) columbic and energy efficiency.


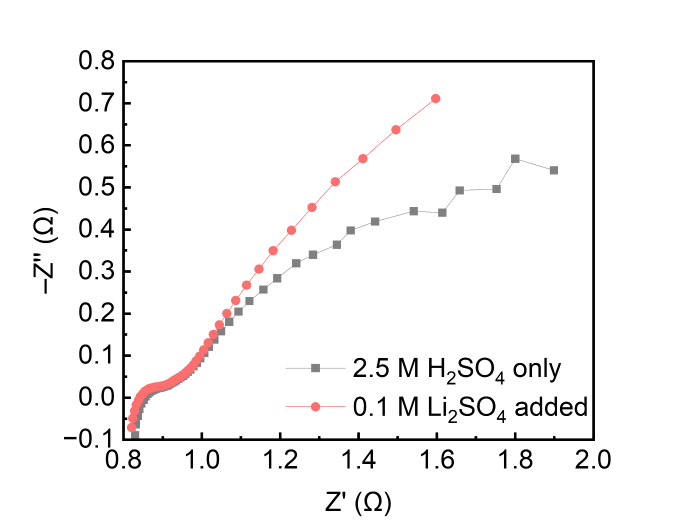


**Figure S22.** The electrochemical impedance spectroscopy (EIS) results of the hybrid Zn-Mn RFB in 2.5 M H_2_SO_4_ supporting electrolyte with and without the addition of Li_2_SO_4_.
